# PHOSPHO1 is a skeletal regulator of insulin resistance and obesity

**DOI:** 10.1186/s12915-020-00880-7

**Published:** 2020-10-22

**Authors:** Karla J. Suchacki, Nicholas M. Morton, Calvin Vary, Carmen Huesa, Manisha C. Yadav, Benjamin J. Thomas, Sophie Turban, Lutz Bunger, Derek Ball, Martin E. Barrios-Llerena, Anyonya R. Guntur, Zohreh Khavandgar, William P. Cawthorn, Mathieu Ferron, Gérard Karsenty, Monzur Murshed, Clifford J. Rosen, Vicky E. MacRae, Jose Luis Millán, Colin Farquharson

**Affiliations:** 1 Roslin Institute, R(D)SVS, University of Edinburgh, Edinburgh, Scotland, UK; 2 Centre for Cardiovascular Science, The Queen’s Medical Research Institute, University of Edinburgh, 47 Little France Crescent, Edinburgh, EH16 4TJ Scotland, UK; 3 Center for Molecular Medicine, Maine Medical Center Research Institute, Scarborough, ME USA; 4 MRC Centre for Reproductive Health, University of Edinburgh, Edinburgh, Scotland, UK; 5 Sanford Burnham Prebys Medical Discovery Institute, La Jolla, USA; 6 Scottish Rural College, Edinburgh, Scotland, UK; 7 Medical Sciences and Nutrition, School of Medicine, University of Aberdeen, Aberdeen, Scotland, UK; 8 International Clinical Research Center, Brno, Czech Republic; 9 Department of Medicine and Faculty of Dentistry, McGill University, Montreal, Canada; 10 Molecular Physiology Research Unit, Institut de recherches cliniques de Montréal, Montreal, Canada; 11 Department of Genetics and Development, Columbia University Medical Center, New York, USA

**Keywords:** PHOSPHO1, Osteocalcin, Choline, Bone, Energy metabolism, Insulin, Endocrine organ, Skeleton, Obesity

## Abstract

**Background:**

The classical functions of the skeleton encompass locomotion, protection and mineral homeostasis. However, cell-specific gene deletions in the mouse and human genetic studies have identified the skeleton as a key endocrine regulator of metabolism. The bone-specific phosphatase, Phosphatase, Orphan 1 (PHOSPHO1), which is indispensable for bone mineralisation, has been recently implicated in the regulation of energy metabolism in humans, but its role in systemic metabolism remains unclear. Here, we probe the mechanism underlying metabolic regulation by analysing Phospho1 mutant mice.

**Results:**

*Phospho1*^*−/−*^ mice exhibited improved basal glucose homeostasis and resisted high-fat-diet-induced weight gain and diabetes. The metabolic protection in *Phospho1*^*−/−*^ mice was manifested in the absence of altered levels of osteocalcin. Osteoblasts isolated from *Phospho1*^*−/−*^ mice were enriched for genes associated with energy metabolism and diabetes; *Phospho1* both directly and indirectly interacted with genes associated with glucose transport and insulin receptor signalling. Canonical thermogenesis via brown adipose tissue did not underlie the metabolic protection observed in adult *Phospho1*^*−/−*^ mice. However, the decreased serum choline levels in *Phospho1*^*−/−*^ mice were normalised by feeding a 2% choline rich diet resulting in a normalisation in insulin sensitivity and fat mass.

**Conclusion:**

We show that mice lacking the bone mineralisation enzyme PHOSPHO1 exhibit improved basal glucose homeostasis and resist high-fat-diet-induced weight gain and diabetes. This study identifies PHOSPHO1 as a potential bone-derived therapeutic target for the treatment of obesity and diabetes.

## Background

In addition to its classical structural functions, the skeleton is a site of significant glucose uptake and is involved in the regulation of whole-body glucose metabolism [1–9]. Osteocalcin (OCN) is the most abundant osteoblast-specific non-collagenous protein derived from bone and is thought to maintain the mechanical properties of the bone matrix by regulating calcium binding when fully carboxylated (GLA13-OCN) [10]. However, when OCN is not γ-carboxylated (uncarboxylated (GLU-OCN) or undercarboxylated (GLU13-OCN)), it is released from bone into the circulation where it is able to regulate whole-body glucose metabolism in an endocrine manner [7, 11–14]. Mice deficient in OCN have increased fat mass and are hyperglycemic, hypoinsulinemic and insulin-resistant in muscle. Furthermore, serum GLU17-OCN (human form of GLU13) levels and β-cell function show an inverse correlation with glycated haemoglobin (*HbA1c*), fat mass and plasma glucose levels [15–19]. Osteoblasts regulate glucose metabolism through OCN-dependent and independent mechanisms [20, 21]. An alternative candidate is the bone-specific cytosolic phosphatase, Phosphatase, Orphan 1 (PHOSPHO1) [22–29]. PHOSPHO1 initiates bone matrix mineralisation, and PHOSPHO1 deficiency causes significant skeletal pathology, bowed long bones, osteomalacia and scoliosis in early life [30–33]. Within the osteoblast, PHOSPHO1 forms choline and inorganic phosphate (Pi) from the hydrolyses of phosphocholine (PCho). The liberated Pi is incorporated into hydroxyapatite during the mineralisation process whereas the choline may alter glucose homeostasis. Elevated levels of choline result in insulin resistance in mice, and choline supplementation induces hyperglycaemia and insulin intolerance in mice via the modulation of plasma glucagon [34].

A number of recent studies have implicated PHOSPHO1 in the regulation of energy metabolism in humans [35–38]. Within the PHOSPHO1 gene, differential methylation sites have been identified as potentially useful biomarkers for clinical application in the early detection of type 2 diabetes [35] and significant associations between methylation at loci within the PHOSPHO1 gene and the future risk of type 2 diabetes exist [36, 37]. Differential methylation in *PHOSPHO1* was associated with three lipid traits (total cholesterol, high-density lipoprotein cholesterol, and triglycerides) [39, 40]. Most recently, genetic variants of *PHOSPHO1 in a* bivariate twin study were found to be associated with body mass index and waist-hip ratio [38]. Taken together, these findings suggest that in addition to the established role of PHOSPHO1 in biomineralisation of the skeleton and dentition, *Phospho1* ablation may result in improved glucose homeostasis and a reduction in metabolic disease susceptibility. We hypothesised that bone-derived choline may be an important regulator of global metabolism. To address this, we examined the metabolic phenotype of juvenile and adult *Phospho1*^*−/−*^ mice.

## Results

### *Phospho1* inactivation improves glucose tolerance and insulin sensitivity in juvenile mice

**Fig. 1:**
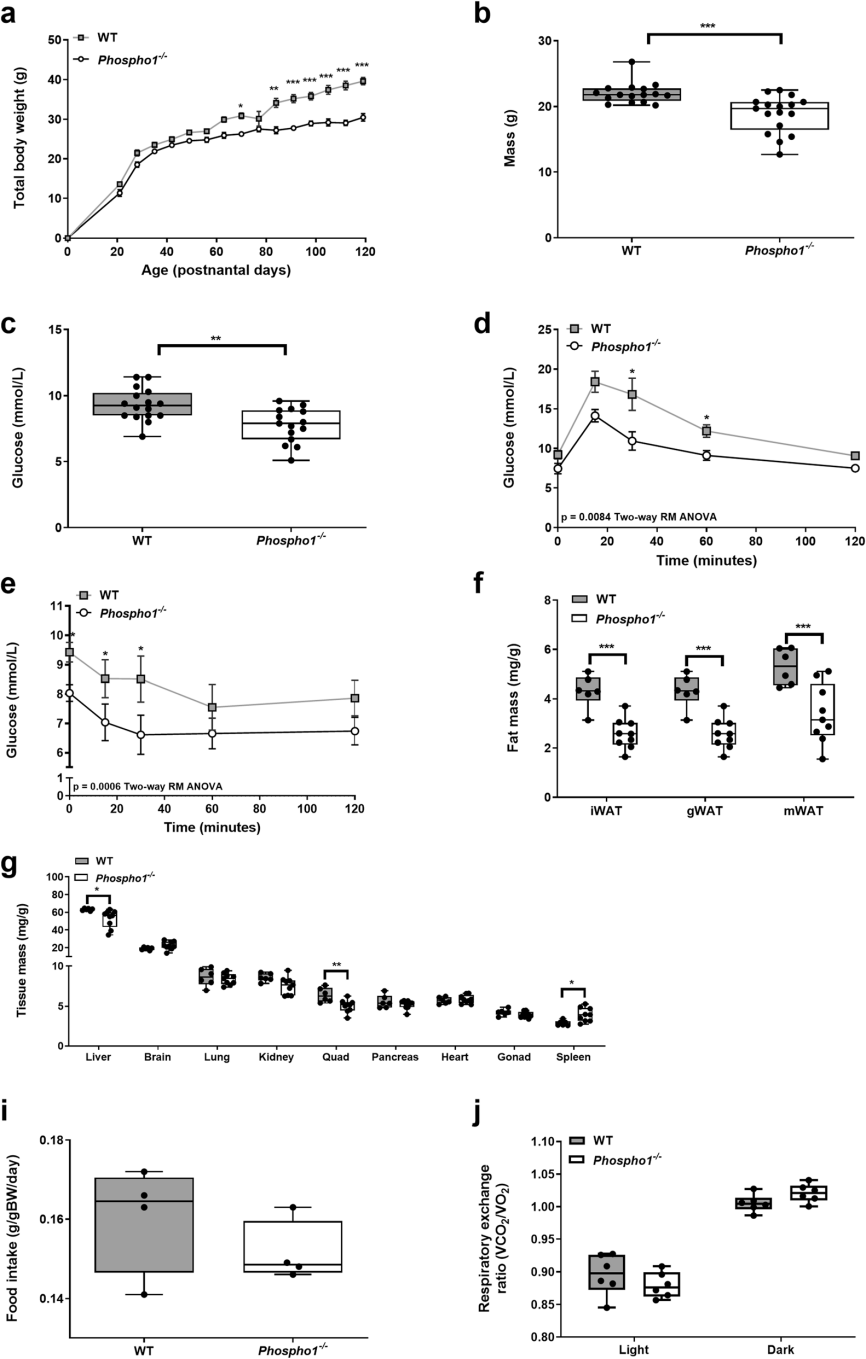
Juvenile *Phospho1*^−/−^ mice display increased insulin sensitivity and decreased fat mass. *Phospho1*^*−/−*^ mice showed decreased (**a**, **b**) growth and live weight at 35 days of age and (**c**) fasting glucose, (**d**, **e**) improved glucose and insulin tolerance and (**f**) decreased adipose tissue. Notable differences in tissue mass were also observed in the liver, quadriceps and spleen. These changes were not a consequence of (**i**) altered food intake or (**j**) energy expenditure. Data in (**a**) and are shown as mean ± SEM of 16–17 mice per group, (**b**) mean ± SEM 6–8 mice per group. *p < 0.05, **p < 0.01, ***p < 0.001 and the data were assessed as follows: (**a**, **d**, **e**) repeated measures two-way ANOVA with Sidak’s multiple comparisons test; (**b**, **c**) two-tailed *t* test; (**f**, **g**) two-tailed unpaired *t* test; **(i**) two-tailed Mann–Whitney test; (**j**) two-tailed unpaired *t* test

Growth of *Phospho1*^*−/−*^ mice was decreased compared to wild-type (WT) mice (Fig. 1a). Juvenile *Phospho1*^−/−^ mice (35-day-old) had reduced body weight and blood glucose levels (WT, 9.31 ± 0.31 mmol/L; *Phospho1*^−/−^, 7.76 ± 0.32 mmol/L; *p* < 0.01) (Fig. 1b, c), improved glucose tolerance (Fig. 1d) and whole body insulin sensitivity compared to WT counterparts (Fig. 1e). Consistent with this, adipose depots were smaller in *Phospho1*^−/−^ mice: inguinal (iWAT; WT, 4.31 ± 0.27 mg/g; *Phospho1*^−/−^, 2.58 ± 0.20 mg/g; *p* < 0.001), mesenteric (mWAT; WT, 5.30 ± 0.30 mg/g; *Phospho1*^−/−^, 3.39 ± 0.40 mg/g; *p* < 0.01) and gonadal (gWAT; WT, 4.31 ± 0.27 mg/g; *Phospho1*^−/−^, 2.58 ± 0.20 mg/g; *p* < 0.001) adipose tissue (Fig. 1f). *Phospho1*^−/−^ mice had significantly smaller livers (WT, 64.36 ± 0.49 mg/g; *Phospho1*^−/−^, 52.71 ± 3.37 mg/g; *p* < 0.05), and quadriceps (WT, 6.39 ± 0.36 mg/g; *Phospho1*^−/−^, 5.01 ± 0.26 mg/g; *p* < 0.01) (Fig. 1g). Food intake (WT, 0.13 ± 0.01 g/gBW/day; *Phospho1*^*−/−*^, 0.12 ± 0.01 g/gBW/day) (Fig. 1i), activity (Additional file 1: Fig. S1) and energy expenditure (day and night respiratory exchange rate (RER)) (Fig. 1j) were comparable between genotypes.

### *Phospho1* deficiency protects from diet-induced diabetes in adult mice

**Fig. 2:**
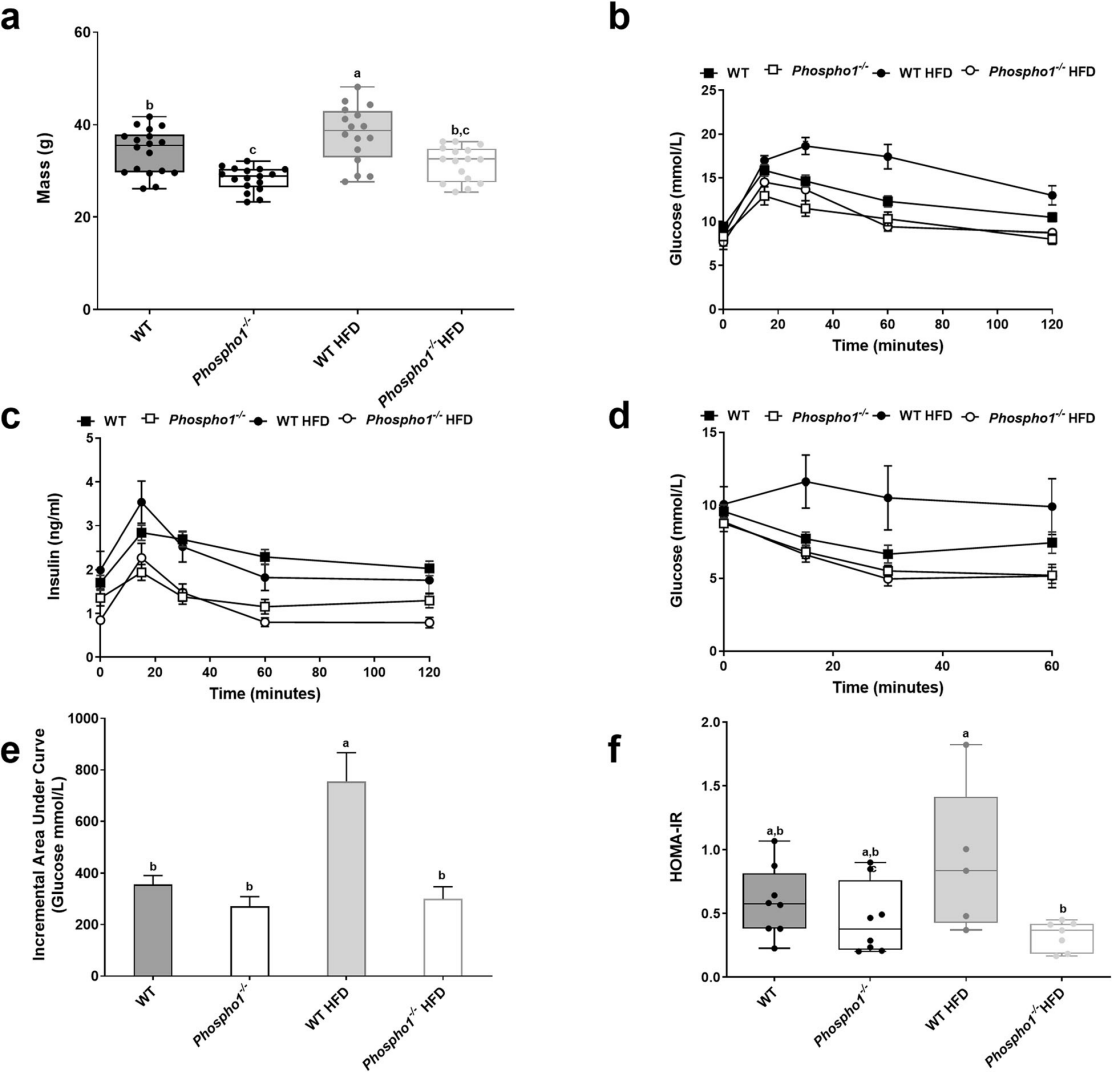
*Phospho1*^−/−^ mice are protected from glucose intolerance. **a** Body mass. **b** Glucose tolerance test (GTT). **c** Glucose stimulate insulin secretion (GSIS). **d** Insulin tolerance test (ITT). **e** Incremental area under the curve for GTT. **f** HOMA-IR. All data are shown as mean ± SEM. **b**–**e** 5–8 mice per group. Different letters above the error bar show significant difference at *p <* 0.05 and the data were assessed as follows: **a**, **e**, **f** Two-way ANOVA with multiple comparisons; **b**–**d** repeated measures two-way ANOVA with Sidak’s multiple comparisons test

We next fed WT and *Phospho1*^−/−^ mice a chronic high fat diet (HFD) from weaning until adulthood (120 days of age). Adult *Phospho1*^−/−^ mice maintained a lower body weight when fed the HFD compared to WT HFD mice (control diet (CD)—WT, 34.20 ± 1.12 g; *Phospho1*^−/−^, 28.30 ± 0.59 g; HFD—WT: 38.0 ± 1.54 g, *Phospho1*^−/−^: 32.4 ± 1.26 g; *p* < 0.05; Fig. 2a). Fasting glucose levels were raised in WT mice fed a HFD but not in *Phospho1*^−/−^ mice (CD—WT, 9.50 ± 0.37 mmol/l; *Phospho1*^−/−^, 8.59 ± 0.27 mmol/l; HFD—WT, 10.3 ± 0.53 mmol/l; *Phospho1*^−/−^, 9.27 ± 0.77) (Additional file 2: Fig. S2).

Glucose tolerance was improved in *Phospho1*^−/−^ mice after chronic HFD (Fig. 2b). Insulin secretion across the glucose tolerance test (GTT) was also lower in *Phospho1*^−/−^ mice on both CD and HFD, suggestive of insulin sensitisation rather than exaggerated β-cell insulin secretion as the major basis of the phenotype (Fig. 2c). This was confirmed with insulin tolerance tests (ITT) after chronic HFD, which revealed greater glucose disposal in *Phospho1*^−/−^ mice (Fig. 2d–f).

### *Phospho1* deficiency protects from diet-induced obesity in adult mice

**Fig. 3:**
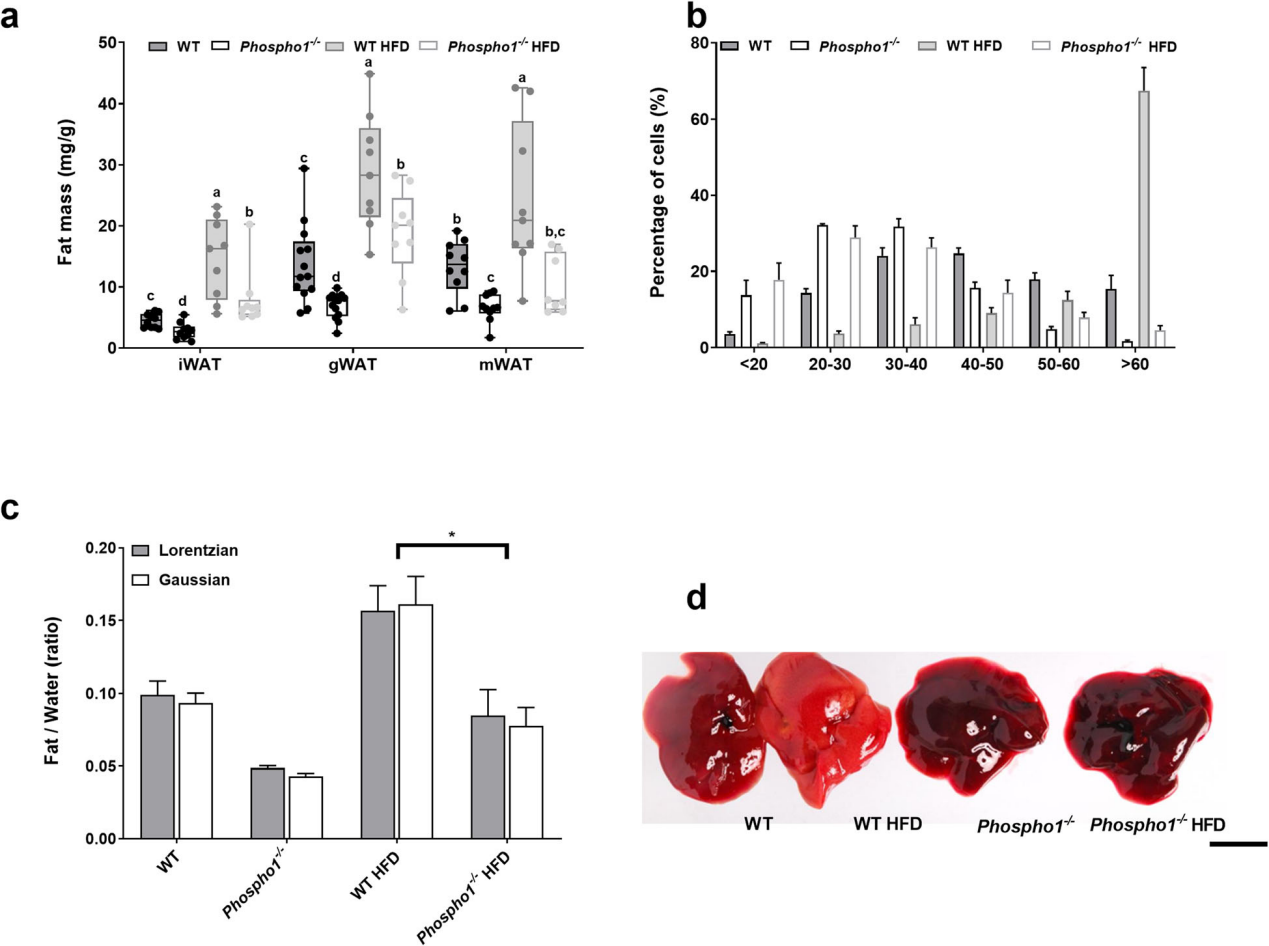
*Phospho1*^−/−^ are protected from NAFLD. **a** Fat analysis of 120-day-old WT and *Phospho1*^*−/−*^ mice on both a control and HFD. **b** Quantification of gonadal fat adipocyte diameter. **c** Quantitative assessment of liver fat utilising spectroscopy. **d** Gross livers of representative mice left to right (WT, WT HFD, *Phospho1*^*−/−*^*, Phospho1*^*−/−*^ HFD; scale bar = 10 mm). All data are represented as mean ± S.E.M. **b**, **c** *n* = 3–4 mice per group. *p < 0.05. Different letters above the error bar show significant difference at *p <* 0.05 and the data were assessed as follows: **a**–**c** Two-way ANOVA with multiple comparisons

The insulin sensitivity observed in *Phospho1*^*−/−*^ mice was consistent with the finding of smaller inguinal (CD—WT, 4.51 ± 0.37 mg/g BW; *Phospho1*^−/−^, 2.79 ± 0.42 mg/g BW; HFD—WT, 14.67 ± 2.12 mg/g BW, *Phospho1*^−/−^, 7.95 ± 1.56 mg/g BW; *p* < 0.01), mesenteric (CD—WT, 13.2 ± 1.34 mg/g BW; *Phospho1*^−/−^, 5.56 ± 1.61 mg/g BW; HFD—WT, 24.14 ± 4.05 mg/g BW; *Phospho1*^−/−^, 10.22 ± 1.57 mg/g BW; *p* < 0.01) and gonadal (CD—WT, 13.7 ± 1.81 mg/g BW; *Phospho1*^−/−^, 6.96 ± 0.58 mg/g BW; HFD—WT, 28.77 ± 3.12 mg/g BW; *Phospho1*^−/−^, 18.78 ± 2.37 mg/g BW; *p* < 0.01) fat depots noted in CD and HFD *Phospho1*^−/−^ mice at necropsy (Fig. 3a). Moreover, confirmation that *Phospho1*^−/−^ mice did not become obese when fed a HFD was shown by μMRI (Additional file 3: Fig. S3). These observations were also not explained by altered activity or increased food intake in 120-day-old adult male mice (data not shown).

Histological analysis revealed smaller gonadal adipocytes in *Phospho1*^−/−^ mice fed both a CD and a HFD. High fat feeding had no significant effect on gonadal adipocyte size in *Phospho1*^−/−^ mice; however, it significantly increased the number of large adipocytes (> 60 μm in diameter) in WT mice (*p* < 0.0001) (Fig. 3b). *Phospho1*^−/−^ mice were also protected from the pronounced hepatic fat accumulation that was noted in WT mice following HFD feeding (Fig. 3c, d).

### Insulin sensitivity in *Phospho1*^*−/−*^ mice is independent of elevated adiponectin serum levels

**Fig. 4:**
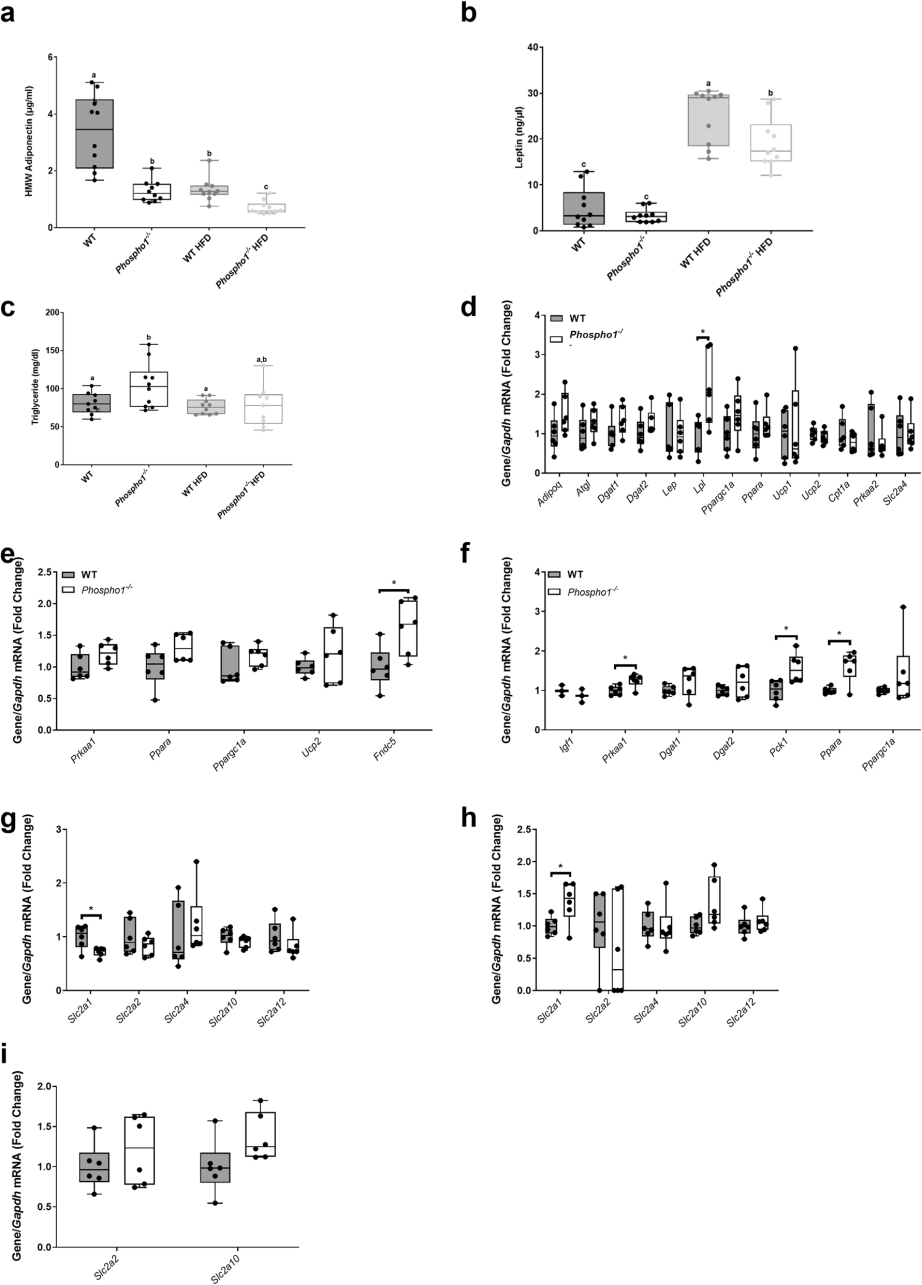
*Phospho1*^*−/−*^ mice are insulin sensitive despite decreased adiponectin. **a** Adiponectin, **b** leptin and **c** triglyceride serum quantification. RT-qPCR analysis of tissue extracted from 120-day-old WT and *Phospho1*^*−/−*^ mice, **d** adipose tissue, **e** quadriceps femoris and **f** liver. RT-qPCR analysis of GLUT receptors from **g** adipose tissue, **h** quadriceps femoris and **i** liver. Data are represented as mean ± S.E.M. * *p* < 0.05. Different letters above the error bar show significant difference at *p <* 0.05 and the data were assessed as follows: **a**–**c** Two-way ANOVA with multiple comparisons, **d**–**i** two-tailed Mann–Whitney test for non-normally distributed data and two-tailed unpaired *t* test for normally distributed data

In an attempt to uncover the mechanism(s) responsible for the increased insulin sensitivity in *Phospho1*^*−/−*^ mice, serum levels of adiponectin and leptin were measured. Levels of high molecular weight adiponectin, a hormone linked to insulin-sensitisation [41], were decreased in *Phospho1*^*−/−*^ mice fed either a CD (2.62-fold) or a HFD (1.92-fold) (both *p* < 0.001) suggesting that insulin sensitivity and protection from obesity are independent of adiponectin (Fig. 4a). The observed decrease in circulating adiponectin in *Phospho1*^*−/−*^ mice was not due to decreased bone marrow adipose tissue, an endocrine organ that contributes significantly to serum adiponectin (Additional file 4: Fig. S4) [42].

Serum leptin levels in CD-fed mice were unaffected by *Phospho1* deficiency, whereas in comparison to WT mice fed a HFD serum leptin levels were significantly decreased 1.31-fold (*p* < 0.05) in *Phospho1*^−/−^ mice fed a HFD (Fig. 4b), accordant with reduced fat mass [43]. *Phospho1*^*−/−*^ CD mice had increased circulating serum triglycerides compared to WT CD mice, but no change was observed in WT and *Phospho1*^−/−^ mice fed a HFD (Fig. 4c). Consistent with increased oxidative metabolism of carbohydrate and lipids in other peripheral tissues, mRNA levels of genes encoding key metabolic proteins were increased in adipose tissue (*Lpl*) muscle (*Fndc5*) and liver (*Prkaa1*, *Pepck1* and *Ppara*) (Fig. 4d-f). The mRNA levels of genes encoding GLUT receptors (Slc2a1, 2, 4, 10 and 12) were largely unchanged (Fig. 4g-i).

### Canonical thermogenesis does not underlie the metabolic protection observed in adult *Phospho1* deficient mice

**Fig. 5:**
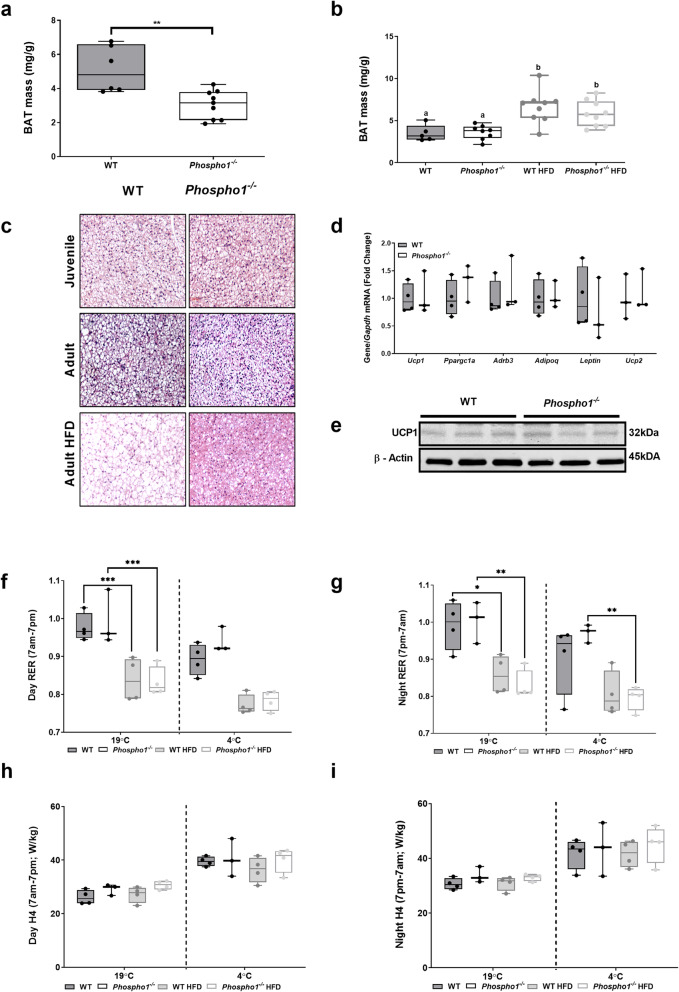
Canonical thermogenesis does not underlie the metabolic protection observed in the adult *Phospho1*-deficient mice. Brown adipose tissue (BAT) mass in **a** juvenile (35-day-old) and **b** adult (120-day-old) WT and *Phospho1*^−/−^ mice. **c** Representative micrographs of BAT from WT and *Phospho1*^−/−^ mice. **d** Brown fat gene expression and **e** UCP1 protein analysis. **f**–**i** Insulin sensitivity and protection from diet induced obesity in *Phospho1*^−/−^ mice was not a consequence of altered energy expenditure (RER—respiratory exchange ratio; H4 = H3 (W)/lean mass (kg)). Data are represented as mean ± S.E.M. *p < 0.05, **p < 0.01, ***p < 0.001. Different letters above the error bar show significant difference at p *<* 0.05 and the data were assessed as follows: **a**, **d** two-tailed Mann–Whitney test. **b**, **d**, **f**–**i** Two-way ANOVA with multiple comparisons

Thirty-five-day-old *Phospho1*^−/−^ mice had decreased interscapular brown adipose tissue (BAT) mass compared to WT counterparts (WT, 5.11 ± 0.57 mg/g; *Phospho1*^−/−^, 3.05 ± 0.40 mg/g; *p* < 0.01) (Fig. 5a). However this reduction in BAT mass did not persist to adulthood nor during high-fat feeding (Fig. 5b). Strikingly, adult and high-fat-fed *Phospho1*^−/−^ mice had smaller brown adipocytes compared to WT controls (Fig. 5c). In order to see if BAT activation and thermogenesis might be responsible for the observed phenotype, we measured key brown fat genes including uncoupling protein 1 (*Ucp1*) (Fig. 5d, e); no differences were observed in the mRNA and protein levels. Furthermore, there were no significant differences in respiratory exchange ratio (RER, indicative of metabolic substrate preference) or energy expenditure between WT or *Phospho1*^−/−^ mice fed either a chow or HFD housed at either room temperature or during cold exposure (4 °C) (Fig. 5f-i). These in vivo data show that increased canonical thermogenesis does not underlie the metabolic protection observed in the *Phospho1*-deficient mice so this line of investigation was not pursued further.

### Diabetes mellitus-associated genes are enriched in *Phospho1*^*−/−*^ primary osteoblasts

**Fig. 6:**
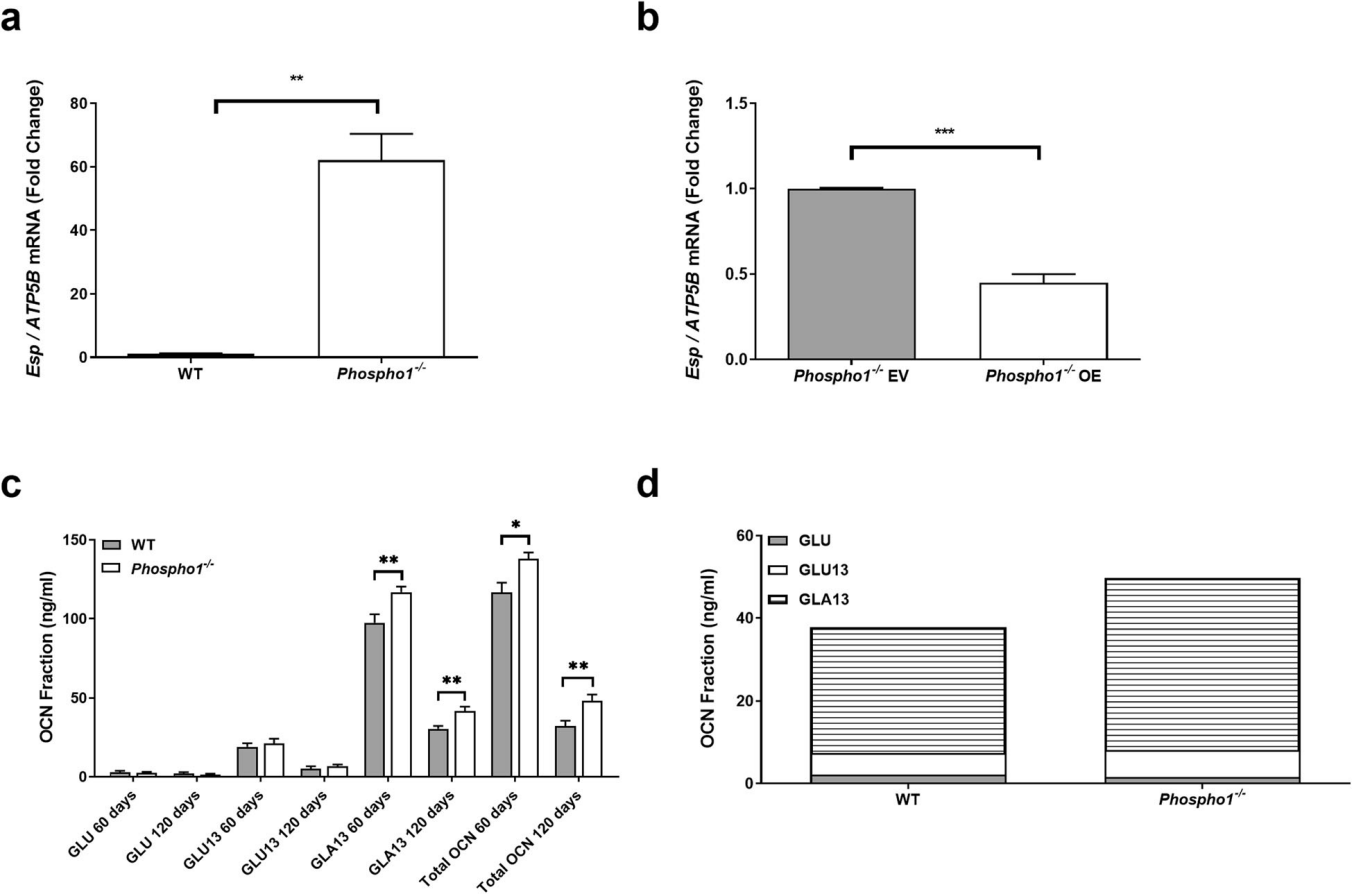
Osteocalcin-independent mechanism of PHOSPHO1-regulated energy metabolism. To assess the relative change in *Esp* mRNA expression in primary calvarial osteoblasts, RT-qPCR was conducted to compare **a** primary WT and *Phospho1*^*−/−*^ osteoblasts and **b** *Phospho1*^*−/−*^ osteoblast transfected with empty (EV) or overexpressing (OE) vectors. **c**, **d** Osteocalcin content of serum from WT and *Phospho1*^*−/−*^ mice at 60 and 120 days of age. Data are represented as mean ± S.E.M *n* = 5–6 mice per group. *p < 0.05, **p < 0.01, ***p < 0.001 and the data were assessed as follows: **a**–**c** Two-tailed Mann–Whitney test or two-tailed unpaired *t* test

We next sought to unravel the genetic circuitry responsible for the improved glucose tolerance in *Phospho1*^***−/−***^ mice. To address this, a transcriptomic analysis of *Phospho1*-deficient osteoblasts was completed. There was a striking 20-fold upregulation of embryonic stem cell phosphatase (*Esp*) mRNA, the gene encoding the protein osteotesticular protein tyrosine phosphatase (OST-PTP) in *Phospho1-*deficient mice [7]. These data were validated in primary WT and *Phospho1*^***−/−***^ calvarial osteoblasts and *Phospho1*-deficient osteoblast overexpressing *Phospho1* (Fig. 6a, b). Protein tyrosine phosphatases are recognised master regulators of insulin receptor signalling (INSR), negatively modifying osteoblast-insulin signalling and thereby controlling GLU13-OCN release [7, 44–48]*.* The identification of elevated *Esp* expression in *Phospho1*^−/−^ mice was strongly suggestive of a reciprocal regulation between OST-PTP and PHOSPHO1 in the control of glucose homeostasis. This increased *Esp* expression was however inconsistent with the improved glucose tolerance in the *Phospho1*^−/−^ mice. Therefore in an attempt to reconcile this anomaly, we measured circulating GLU–OCN and GLU13-OCN, which were found to be unchanged in juvenile and adult *Phospho1*^*−/−*^ mice (Fig. 6c). Only GLU–OCN and GLU13-OCN present in the serum have been shown to act as a hormone [5, 49]. Therefore, these data implied that elevated serum levels of uncarboxylated or undercarboxylated OCN did not mediate the improved metabolic phenotype (Fig. 6c, d). The elevated levels of carboxylated and total OCN in *Phospho1* deficiency was consistent with increased bone turnover in these mice as previously reported [30].

### *Phospho1*^−/−^ calvarial osteoblasts show a greater metabolic capacity in utilising exogenous substrates compared to WT osteoblasts

Primary calvarial osteoblast metabolism analysis revealed that differentiated *Phospho1*^*−/−*^ osteoblasts had elevated basal and FCCP (protonophoric uncoupler) induced oxygen consumption rates (indicative of oxidative phosphorylation) compared to WT osteoblasts when supplied exogenously with glucose, pyruvate and glutamine (Additional file 5: Fig. S5). There was a significant increase in the glycolytic rate of both the non-differentiated and differentiated *Phospho1*^−/−^ osteoblasts compared to WT osteoblasts suggesting that they have increased glucose metabolism. Based on these observations, we conclude that differentiating *Phospho1*^−/−^ calvarial osteoblasts show a greater metabolic capacity in utilising exogenous substrates compared to WT osteoblasts.

**Fig. 7:**
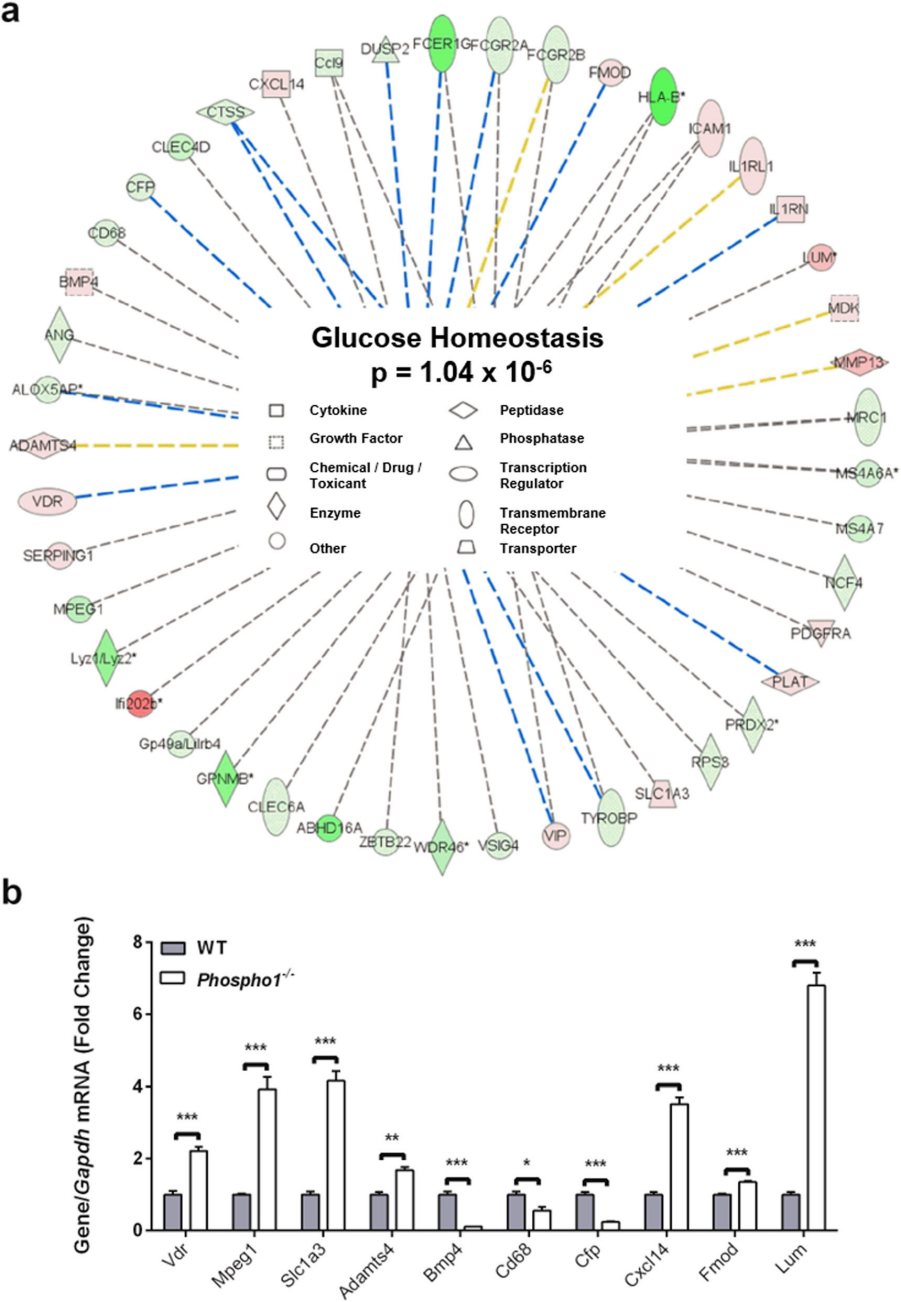
Ingenuity Pathways Analysis network summary predictions. **a** Ingenuity Pathways Analysis was used to predict further genes associated with glucose homeostasis based upon the 22 genes found to be differentially expressed in the microarray. **b** Predicated genes were analysed by RT-qPCR in WT and *Phospho1*^*−/−*^ primary calvarial osteoblast’s. Results were normalised to the *Atp5b* housekeeping gene. Data are represented as mean ± S.E.M (*n* = 3 replicates). *P < 0.05, **P < 0.01, ***P < 0.001. Red = Upregulated. Green = Downregulated (the darker the shade of green and red colour indicates a more extreme up/down regulation, conversely the paler the shade indicates a more subtle up/down regulation. Dashed line = indirect interaction (blue = inhibition, yellow = findings underlying the relationship are inconsistent with the state of the downstream node, grey = Ingenuity Pathways Analysis prediction) and the data were assessed in (**b**) using a two-tailed unpaired *t* test

To further clarify the genetic pathways underpinning these observations, 22 differentially expressed genes identified from the microarray analysis were found by Ingenuity Pathway Analysis (IPA) to be associated with glucose homeostasis (Additional file 6: Table. S6). Further predictive analysis of the differentially expressed genes of the original microarray data set identified > 40 genes to be associated with energy metabolism (*p* = 1.04 × 10^−6^) (Fig. 7a) of which 10 were found to be associated with both diabetes and bone, following an NCBI (*PubMed*) in silico search. Validation by RT-qPCR confirmed that 70% of the genes predicted by IPA (*Vdr*, *Slc1a3*, *Adamts4*, *Cd68*, *Cfp*, *Fmod* and *Lum*) were differentially regulated in *Phospho1*-deficient osteoblasts (*p* < 0.05; Fig. 7b and Additional file 7: Table. S7). Furthermore, GeneMANIA network analysis predicted that *Phospho1* both directly and indirectly interacts with 36 genes associated with glucose transport and metabolic processes and insulin receptor signalling (Additional file 8: Fig. S8). Of the output genes, *Atf4*, *Foxo1* and *Insr* are recognised skeletal modulators of energy metabolism, suggesting crosstalk between *Phospho1* and other metabolic regulatory genes [5, 50, 51].

### Identification of differentially expressed serum proteins in *Phospho1*^*−/−*^ mice

To explore the secretome profile, quantitative SWATH (sequential window acquisition of all theoretical spectra) MS (mass spectrometry) proteomics [52] was conducted on serum from WT and *Phospho1*^*−/−*^ mice fed CD and HFD. Differentially expressed proteins (> 100) were identified in HFD *Phospho1*^*−/−*^ serum compared to HFD-fed WT mice. These proteins were highly associated with glycolysis, gluconeogenesis and ‘metabolic pathways’ (Additional file 9: Table. S9). Pathway and network analysis predicted that the identified proteins interacted with miR-34a, a microRNA that is known to affect diverse parts of insulin signalling in the pancreas, liver, muscle and adipose tissue [53].

### Bone-derived choline is involved in global energy regulation

**Fig. 8:**
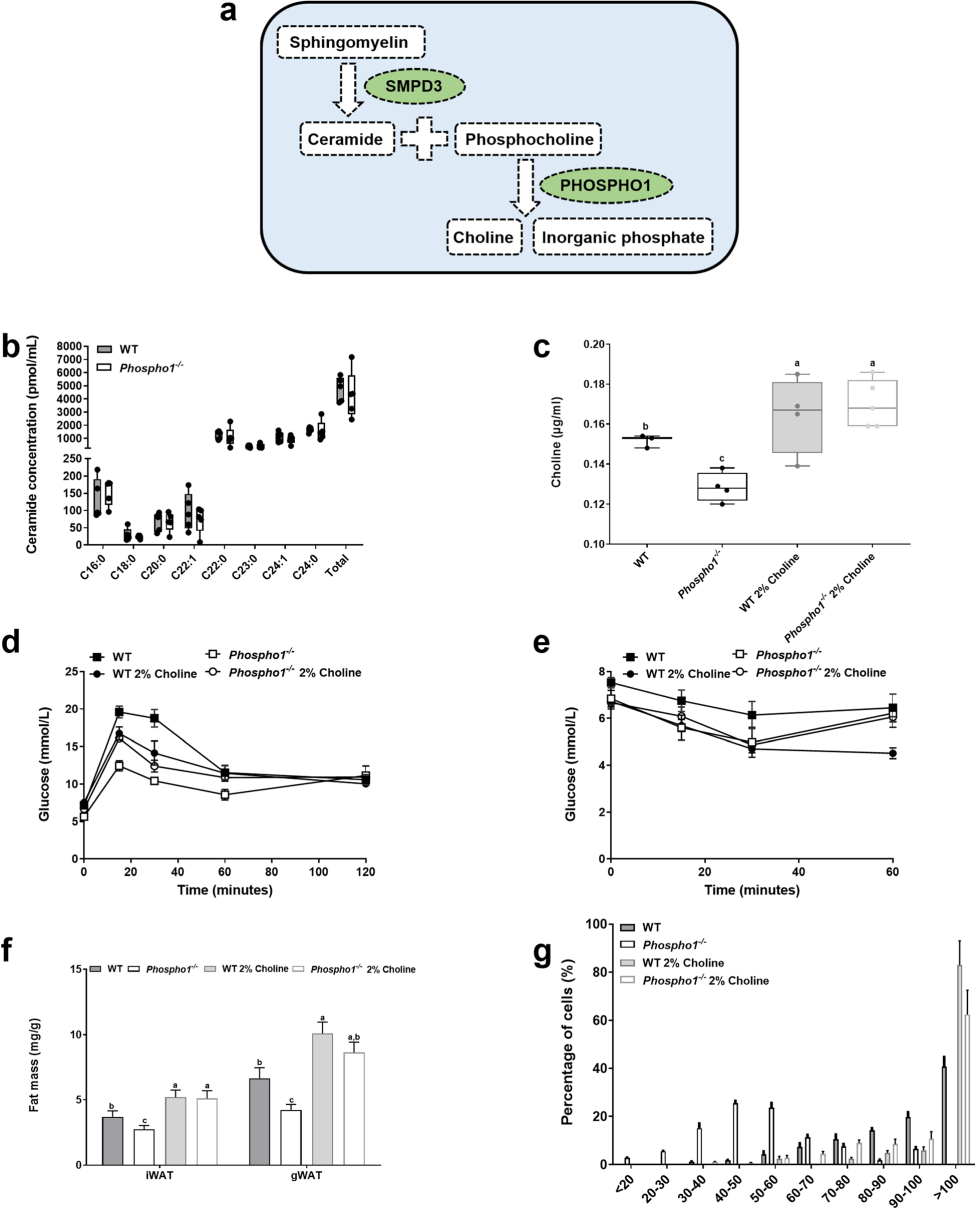
Bone derived choline regulates insulin sensitivity. **a** Schematic diagram outlining the mechanisms by which ceramide and choline and linked. **b** Mouse serum ceramide and choline (**c**) analysis by LC-MS/MS (ceramide) and assay (choline). **d** GTT. **e** ITT. **f** Dissected fat depot weights. **g** Quantification of epididymal fat adipocyte diameter and representative histology. Data are represented as mean ± S.E.M (**c**–**g**) *n* = 3–5 per group. Different letters above the error bar show significant difference at *p <* 0.05 and the data were assessed as follows: **c**, **f**, **g** Two-way ANOVA with multiple comparisons. **b** One-way ANOVA with Dunnet’s or Tukey’s tests for multiple comparisons. **d**, **e** repeated measures two-way ANOVA with Sidak’s multiple comparisons test

Neutral sphingomyelinase 2 (nSMase2) catalyses the hydrolysis of sphingomyelin to form ceramide and PCho [54]. Furthermore, PCho is the preferred substrate for PHOSPHO1 yielding choline and Pi (Fig. 8a) [55]. As elevated levels of both ceramide and choline result in insulin resistance in mice [34, 56] we targeted these intermediates to establish if alterations in their serum levels could explain the insulin sensitive phenotype in *Phospho1*^*−/−*^ mice [34, 56]. The levels of various ceramide species were unchanged (Fig. 8b); however, *Phospho1*^*−/−*^ mice had a significant decrease in serum choline levels (WT, 0.152 ± 0.001 μg/ml; *Phospho1*^−/−^, 0.128 ± 0.003 μg/ml; *p* < 0.01) (Fig. 8c) which were normalised upon choline supplementation. Supplementation of WT and *Phospho1*^*−/−*^ mice with a 2% choline diet (a well-tolerated, palatable diet [57]), also normalised the insulin sensitivity measured in *Phospho1*^*−/−*^, measured by GTT (Fig. 8d). However, unlike WT mice which when fed a 2% choline diet took longer to recover from the insulin challenge, *Phospho1*^*−/−*^ showed no metabolic change in response to insulin between the diets (Fig. 8e). Furthermore, choline supplementation normalised the lean phenotype observed in *Phospho1*^*−/−*^ mice (Fig. 8f, g). These results support the notion that *Phospho1* deficiency improves the metabolic profile of mice in vivo and confers resistance to obesity and diabetes in part via the alteration of serum choline levels.

## Discussion

The fundamental observations presented in this study further strengthen the concept that the skeleton acts as an endocrine organ and provides empirical evidence for a critical role for PHOSPHO1 in energy metabolism. *Phospho1* deficiency results in decreased blood glucose levels, improved insulin sensitivity, glucose tolerance and conferred protection from diet-induced obesity and diabetes in mice despite a 60-fold upregulation of *Esp* expression by *Phospho1*^−/−^ osteoblasts. Mice lacking *Esp* in osteoblasts present with severe hypoglycaemia and hyperinsulinemia resulting in postnatal lethality in the first 2 weeks of life [7]. Conversely, mice overexpressing *Esp* exclusively in osteoblasts were glucose intolerant and insulin resistant [7, 44–48]. Intriguingly, the increased insulin sensitivity in *Phospho1*^*−/−*^ mice was not associated with the expected rise in serum GLU13-OCN levels suggesting that PHOSPHO1-regulated energy metabolism is via OCN-independent mechanisms. This notion has previously been observed when partial genetic ablation of osteoblasts profoundly affected energy expenditure, gonadal fat weight and insulin sensitivity which were not restored by the administration of OCN [20, 21]. Nevertheless, it is possible that the increased insulin sensitivity noted in *Phospho1*^−/−^ mice may be primed by an initial rise in GLU13-OCN levels, which is eventually normalised in a compensatory manner by the observed increase in *Esp* expression. This being the case we would predict that the loss of *Esp* on a *Phospho1*^*−/−*^ background would exacerbate the insulin sensitivity due to increased GLU13-OCN serum levels. These data strengthen the concept that a novel pathway exists between osteoblasts and glucose homeostasis; however, it does highlight the potential cross-talk between OCN-dependent and OCN-independent mechanisms of glucose metabolism.

The role of PHOSPHO1 in controlling bone mineralisation has been extensively investigated through the use of both in vitro and in vivo mouse models. Crucial for the initiation of mineralisation within matrix vesicles, PHOSPHO1 hydrolyses membrane lipid derivatives, primarily PCho to produce Pi (utilised in hydroxyapatite formation) and choline [55]. Phosphocholine can be formed from choline via choline kinase activity or phosphatidylcholine via PLA2 and ENPP6 as well as from the hydrolysis of sphingomyelin, via nSMase2 to form PCho and ceramide [58]. Mindful of this, it has been reported that elevated levels of both ceramide and choline result in insulin resistance in mice [34, 56]. We saw no change in ceramide species in *Phospho1*^−/−^ mice; however, there was a significant decrease in serum choline levels in *Phospho1*^−/−^ mice, which was normalised in *Phospho1*^−/−^ mice fed a 2% choline rich diet resulting in a normalisation in insulin sensitivity and fat mass. This study highlights for the first time the importance of bone-derived choline in the regulation of energy metabolism; however, it remains clear that choline supplementation alone does not fully correct all metabolic defects in *Phospho1*^−/−^ mice.

The regulation of global energy metabolism by the skeleton is a complex, multifactorial process, and it is therefore likely that presently undefined bone secreted factors may have a significant role in PHOSPHO1’s ability to regulate global energy. We identified > 100 secreted proteins that may contribute to the energy regulation via the skeleton. These unique proteins were highly associated with glycolysis, gluconeogenesis and ‘metabolic pathways’ and association with miR-34a, a microRNA that affects diverse parts of insulin signalling in the pancreas, liver, muscle and adipose tissue [53, 59]. Furthermore, lumican, a proteoglycan secreted by differentiating osteoblasts and a constituent of the bone matrix was found to be enriched in serum of *Phospho1*^*−/−*^ mice by both proteomic and microarray analysis. Interestingly, lumican has also been observed in the decidua of diabetic patients [60, 61]. Further investigations of these and other candidates may uncover new skeletal regulators of energy metabolism that act via osteocalcin independent mechanisms.

The metabolic phenotype observed in the *Phospho1*-deficient mice was not due to increased BAT activation despite striking histological differences between WT and *Phospho1*^*−/−*^ tissue. BAT is a thermogenic organ that increases energy expenditure to generate heat, maintaining body temperature in a cold environment [62]. When activated by cold exposure or through *activation* of *β3*-adrenergic receptors, BAT improves insulin sensitivity and lipid clearance, highlighting its key role in metabolic health [63]. PHOSPHO1 has been previously suggested to have a role in murine BAT function. Increased expression of *Phospho1* in both isolated brown and white adipocytes was observed following treatment with the β-adrenergic agonist (CL316,243) compared to placebo-treated white adipocytes [64]. Mice with an adipose-specific defect in fatty acid oxidation (*Cpt2*^*A−/−*^) showed a loss of β3-adrenergic induced *Phospho1* expression in BAT [64] and expression of *Phospho1* was shown to be elevated in *Ucp1*-deficient animals; however, WT mice had very low *Phospho1* expression in BAT and no detectable PHOSPHO1 was observed in WT iWAT and BAT [65]. No difference in *Phospho1* gene expression was observed between human supraclavicular and subcutaneous adipose progenitor cells (GEO Dataset: GDS5171/8016540) and murine WAT had elevated *Phospho1* gene expression compared to BAT (GEO Dataset: GDS2813/1452485_at). In our hands, we have noted high expression of *Phospho1* in murine BAT but no protein expression was detected (Additional file 10: Fig. S10). Taken together, these data suggest that *Phospho1* is likely to play a role in BAT function; however, the BAT phenotype observed in *Phospho1* deficient mice does not appear to underlay the metabolic protection we see in these animals. Further studies are necessary to unravel the role of PHOSPHO1 in BAT using a BAT conditional knock-out model of *Phospho1.*

## Conclusions

Collectively, the results of this study add further credibility to the concept that GLU13-OCN is not the sole mediator of the endocrine function of the skeleton [20]. We suggest, as others have, that further undefined bone-derived proteins/lipids work in partnership with OCN to regulate the metabolic function of the skeleton and affect other metabolic organs such as muscle and liver [20]. Indeed, this study has identified other potential protein mediators and raised the possibility that bone-derived choline may contribute to the regulation of the development of the metabolic syndrome. Furthermore, our results suggest that *Esp* may act as a fine controller of insulin sensitivity in mice, offering protection from severe hypoglycaemia and dyslipidaemia.

Several previous reports have suggested an association between PHOSPHO1 expression in disorders of altered energy metabolism such as obesity and diabetes [35–39]. The data from this present study is both supportive of such an association but also provides insight into the mechanisms by which PHOSPHO1 may contribute to the regulation of energy metabolism, inclusive of insulin sensitivity, glucose tolerance and fat metabolism. Also, inhibitors of PHOSPHO1 activity such as the proton pump inhibitor lansoprazole, commonly prescribed to control and prevent symptoms of gastroesophageal reflux disease and dyspepsia, have been associated with improved glycaemic control in diabetic patients [28, 66–68]. Taken together, the identification of PHOSPHO1 in the role of energy metabolism in both the human and mouse offers the potential to manipulate key targets of the PHOSPHO1 pathway to improve metabolic health [36, 37].

## Methods

### Aim

Examine glucose metabolism and the energy status of *Phospho1*^*−/−*^ mice.

### Reagents

All chemicals, tissue culture medium and buffers were from Sigma-Aldrich (Dorset, UK) and Invitrogen (Paisley, UK) unless otherwise stated. PCR oligonucleotides were purchased from MWG Eurofins (Ebersberg, Germany) and Primer Design (Southampton, UK). PHOSPHO1 HuCAL Fab bivalent antibody was purchased from AbD Serotech (Kidlington, UK). All antibodies were diluted 1:1000 unless otherwise noted.

### Animals

*Phospho1* null mice were generated as previously described [31]. Offspring carrying the mutant *Phospho1* gene were identified by genotyping (F: 5′-TCCTCCTCACCTTCGACTTC-3′, R: 5′-TCCTCCTCACCTTCGACTTC-3′). All in vivo studies were conducted at 120 days of age unless otherwise stated. Male mice were fed a high fat diet consisting of 58% of calories from fat (DBM Scotland, Broxburn, UK) or control diet (6.2% calories from fat; Harlan Laboratories, Indianapolis, IN, USA) starting at 4 weeks of age. Male mice were fed a 2% supplemented choline diet (Harlan Laboratories) or control diet (Harlan Laboratories) for 5 weeks prior to cull at 120 days. Ad libitum food consumption was monitored for 6 days and basal nocturnal activity was quantified using an AM524 Single Layer X, Y IR activity monitor and associated Amonlite software (Linton Instrumentations, Norfolk, UK). Juvenile metabolic activity was measured using indirect calorimetry (Oxymax Lab Animal Monitoring System: CLAMS (Columbus Instruments, OH USA). Adult metabolic rate was measured using indirect calorimetry (TSE PhenoMaster 1.0, with software version 6.1.9). Cold exposed mice were first housed in these cages for 3 days at room temperature (RT) for acclimation and baseline measurements. Mice were then house for 72 h at 4 °C. All experiments were conducted blind to the operator. Animals were maintained under conventional housing conditions with a 12-h light/dark cycle with free access to food and water (except when food was restricted during fasting). All animal experiments were approved by The Roslin Institute’s Animal Users Committee, and the animals were maintained in accordance with UK Home Office guidelines for the care and use of laboratory animals.

### Metabolic studies

Male juvenile and adult (35 and 120 days old respectively) were weighed and fasted for 4 h between 9 am and 1 pm. Prior to the start of the tests, a basal blood sample was collected by venesection into EDTA powder-coated capillary tubes (Starstedt, Leicester, UK). Basal glucose levels were measured using a glucose monitoring system (Accu-Chek® Aviva, Roche, Leicester, UK). Two milligrammes of D-glucose (Sigma, Poole, UK) per g of body weight was administered by gavage or 0.5 mU of insulin (Actrapid, NovoNordisk, Bagsvaerd, Denmark) per g/body weight was administered intraperitoneally (i.p.). At precisely 15, 30, 60 and 120 min following administration, blood glucose was measured with an Accu-Chek® Aviva glucose meter (Roche Diagnostics Ltd., Lewes, UK) and insulin was measured by ELISA (ChrystalChem, Chicago, IL, USA). Animals were allowed to recover for 2 weeks prior to euthanasia. Tissues were collected for protein, gene and histological analysis.

### Serum measurements

Serum samples were prepared from blood collected by heart puncture of CO_2_ culled mice in the fasted state. Total, carboxylated (GLA13-OCN), undercarboxylated (GLU13-OCN) and uncarboxylated (GLU-OCN) osteocalcin [49], adiponectin and leptin (CrystalChem) were quantified by ELISA.

### Primary osteoblast isolation and culture

Under sterile conditions, calvaria were isolated from 2- to 4-day-old new-born WT and *Phospho1*^*−/−*^ mice as previously described [69]. Osteoblasts were expanded in flasks in growth medium consisting of α-MEM supplemented with 10% FBS and 1% gentamicin in a humidified atmosphere of 95% air/5% CO_2_ and maintained at 37 °C. When the cells reached 80–90% confluency, they were seeded at a density of 2.5 × 10^4^/cm^2^ in multi-well plates. Conditioned medium was collected upon plate confluency, centrifuged to remove particulates and frozen at − 80 °C until required. For overexpression studies, primary osteoblasts were transfected with empty (EV) or overexpressing (OE) vectors as previously described [70].

### Mito stress test

An XF24 Analyser (Agilent Technologies, Santa Clara, CA, USA) was used to measure the respiratory function of primary osteoblasts. Osteoblasts were plated at a density of 50,000 cells per well and transferred to a 37 °C CO_2_ incubator until and calvarial osteoblasts were differentiated using osteogenic differentiation media containing 8 mM β-glycerophosphate and 50 μg/ml ascorbic acid for 3 days. On the day of the assay, cells were washed in XF Assay Media supplemented with 25 mM glucose and 10 mM pyruvate and placed in a non-CO2 incubator at 37 °C for 1 h prior to start of assay. Reagents were prepared for the assay (injection volume of 75 μL for each reagent per well) from 2.5 mM Seahorse stock solutions, Oligomycin (1.2 μM). Following equilibration, the Seahorse plate was placed in the Seahorse XF24 Analyser for sample analysis. The raw data was normalised to protein content in each well at the end of the assay [71].

### Gene expression analyses and immunoblotting

RNA extractions from tissues and cells were performed using the RNeasy Lipid Tissue Kit (Qiagen). The SuperScript First Strand Synthesis System (Invitrogen) was used for reverse transcription. Real-time PCR amplification with the 2 x precision master mix (Primer design, Southampton, UK) using the Stratagene Mx3000P real-time QPCR system (Agilent Technologies, Santa Clara, CA, USA). Each sample was tested in triplicate and compared to a housekeeping gene (Atp5B in osteoblasts and bone tissue, Lrp10 in adipose tissue and Gapdh in all other tissues) using MxPro software (Cheshire, UK), and the relative expression of the analysed genes was calculated using the ΔΔCT method [72]. All primer sequences are described in Table 1. Primers sequences are available in Table 1. For protein extraction, cells were scraped and tissues homogenised in an appropriate volume of radio-immunoprecipitation assay (RIPA) buffer containing 15% of complete mini protease inhibitor cocktail (Roche, Burgess Hill, West Sussex, UK). Protein concentration was determined by the Bio-Rad DC protein assay (Bio-Rad, Hertfordshire, UK). Immunoblotting was conducted with specific antibodies and protein bands were visualised using the enhanced chemiluminescence (ECL) Western Blotting Detection System (GE Healthcare, Chalfont St Giles, UK) or the Odyssey infrared detection system (LICOR). All antibodies are described in Table 2.

**Table 1:** Sequences of primers used for qPCR

Species	Transcript	Primer sequences 5′ to 3′
*M. musculus*	*Adamts4*	F: ATGGCCTCAATCCATCCCAG
		R: GCAAGCAGGGTTGGAATCTTTG
*M. musculus*	*Adipoq*	F: GGCCGTTCTCTTCACCTACG
		R: TGGAGGAGCACAGAGCCAG
*M. musculus*	*Adrb3*	Primer Design, Southampton, UK; sequence not disclosed
*M. musculus*	*Atgl*	Primer Design, Southampton, UK; sequence not disclosed
*M. musculus*	*Atp5b*	Primer Design, Southampton, UK; sequence not disclosed
*M. musculus*	*Bmp4*	F: ATTCCTGGTAACCGAATGCTG
		R: CCGGTCTCAGGTATCAAACTAGC
*M. musculus*	*Cd68*	F: CCATCCTTCACGATGACACCT
		R: GGCAGGGTTATGAGTGACAGTT
*M. musculus*	*Cfp*	F: CTGCTACTGGTTATCCTGCCA
		R: TCTACCCTGATGTCTCTCCCA
*M. musculus*	*Cpt1a*	Primer Design, Southampton, UK; sequence not disclosed
*M. musculus*	*Cxcl14*	Primer Design, Southampton, UK; sequence not disclosed
*M. musculus*	*Dgat1*	Primer Design, Southampton, UK; sequence not disclosed
*M. musculus*	*Dgat2*	Primer Design, Southampton, UK; sequence not disclosed
*M. musculus*	*Esp*	Primer Design, Southampton, UK; sequence not disclosed
*M. musculus*	*Fmod*	Primer Design, Southampton, UK; sequence not disclosed
*M. musculus*	*Fndc5*	Primer Design, Southampton, UK; sequence not disclosed
*M. musculus*	*Igf1*	Primer Design, Southampton, UK; sequence not disclosed
*M. musculus*	*Lep*	F: ATTTCACACACGCAGTCGGTAT
		R: GGTGGAGCCCAGGAATGAAG
*M. musculus*	*Lpl*	F: TCTGTACGGCACAGTGG
		R: CCTCTCGATGACGAAGC
*M. musculus*	*Lum*	F: CTCTTGCCTTGGCATTAGTCG
		R: GGTCATCACAGTACATGGCAGT
*M. musculus*	*Mpeg1*	F: CTGGATGATAATAGCGTGTGCT
		R: AAGACAGGTAGTTTCAGGGCA
*M. musculus*	*Pck1*	F: CATATGCTGATCCTGGGCATAAC
		R: CAAACTTCATCCAGGCAATGTC
*M. musculus*	*Phospho1*	F: TTCTCATTTCGGATGCCAACA
		R: TGAGGATGCGGCGGAATAA
*M. musculus*	*Ppara*	F: CCCTGAACATCGAGTGTCGA
		R: AATAGTTCGCCGAAAGAAGCC
*M. musculus*	*Ppargc1a*	F: CCCTGCCATTGTTAAGACC
		R: TGCTGCTGTTCCTGTTTTC
*M. musculus*	*Prkaa1*	Primer Design, Southampton, UK; sequence not disclosed
*M. musculus*	*Prkaa2*	F: GTCAAAGCCGACCCAATGATA
		R: CGTACACGCAAATAATAGGGGTT
*M. musculus*	*Slc1a3*	F: ACCAAAAGCAACGGAGAAGAG
		R: GGCATTCCGAAACAGGTAACTC
*M. musculus*	*Slc2a1*	F: TCAACACGGCCTTCACTG
		R: CACGATGCTCAGATAGGACATC
*M. musculus*	*Slc2a10*	F: ACCAAAGGACAGTCTTTAGCTG
		R: ATCTTCCAAGCAGACGGATG
*M. musculus*	*Slc2a12*	F: GGGTGTCAACCTTCTCATCTC
		R: CCAAAGAGCATCCCTTAGTCTC
*M. musculus*	*Slc2a2*	F: TGTGCTGCTGGATAAATTCGCCTG
		R: AACCATGAACCAAGGGATTGGACC
*M. musculus*	*Slc2a4*	F: CCAGTATGTTGCGGATGCTAT
		R: TTTTAGGAAGGTGAAGATGAAGAAG
*M. musculus*	*Ucp1*	F: GGATGGTGAACCCGACAACT
		R: AACTCCGGCTGAGAAGATCTTG
*M. musculus*	*Vdr*	F: GAATGTGCCTCGGATCTGTGG
		R: ATGCGGCAATCTCCATTGAAG

**Table 2:** Antibodies used for Western blotting

Protein	Species	Source	Catalogue Number	Dilution	Band size (kDa)
PHOSPHO1	Human	AbD Serotec	HCA093	1:1000	30
UCP1	Rabbit	Cell Signaling	#14670	1:1000	32
β-actin (HRP-linked)	Mouse	Sigma	A3854	1:50,000	45

### Tissue histology

Tissue was fixed in 4% PFA and embedded in paraffin wax. Five-micrometre sections were stained with haematoxylin and eosin (H&E) using the Leica Autostainer and mounted in DePeX (VWR, Lutterworth, UK). Adipocyte diameter and pancreatic β-cell islet number and size were quantified using ImageJ software as previously described [7, 73].

### Osmium staining

Mouse tibiae were fixed in 10% neutral-buffered formalin and decalcified in 14% EDTA, pH 7.4. Mouse bones were stained according to [74]. Briefly, bones were stained with a 1% osmium tetroxide solution for 48 h at room temperature. Bones were washed in Sorensen’s buffer and embedded in 1% agarose prior to μCT scanning (μCT100 Scanco Medical, Bassersdorf, Switzerland) − 12 μm, medium resolution, 70 kVp, 114 μA, 0.5 mm AL filter and integration time 500 ms. Analysis was performed using the manufacturer’s software.

### Micro-magnetic resonance, computed tomography and liver spectroscopy

CD and HFD mice were sacrificed immediately before imaging. For micro-magnetic resonance, mice were imaged on a Varian 7 Tesla magnet using VnmrJ Pre-Clinical MRI Software. T2-weighted images were acquired both in the axial (1 mm thickness, 192 × 192 pixels, TR – 3000 ms, TE 24 ms, 1 average, FOV – 38.4 × 38.4) and coronal planes (0.5 mm thickness, 512 × 256 pixels, TR – 3000 ms, TE 24 ms, 4 averages, FOV – 102.4 × 51.2). Liver spectroscopy was conducted on user defined areas (TR - 1800 ms, TE – 11.5 ms, 16 averages, Vauxhall 3x3x3). Lorentzian and Gaussian lineshape were used to fit peaks to MR data (jMRUI http://www.mrui.uab.es/mrui/mrui).

### Microarray and pathway analysis

Labelled cRNA was prepared from 500 ng of WT and *Phospho1*^*−/−*^ primary calvarial osteoblast RNA using the Illumina® RNA Amplification Kit from Ambion (Austin, TX, USA). The labelled cRNA (1500 ng for mouse and 750 ng for human) was hybridised overnight at 58 °C to the SentrixMouseWG-6 Expression BeadChip or humanHT-12 Expression BeadChip (> 46,000 gene transcripts; Illumina, San Diego, CA, USA) according to the manufacturer’s instructions. BeadChips were subsequently washed and developed with fluorolink streptavidin-Cy3 (GE Healthcare). BeadChips were scanned with an Illumina BeadArray Reader. Data was generated from Imagedata using Illumina software, GenomeStudio. Normalised data was generated using ^3^Cubic Spline^2^ Model in software. Pathway analysis was performed with Ingenuity Pathway Analysis (IPA, Ingenuity® Systems, www.ingenuity.com) and GeneMANIA (http://www.genemania.org).

### Proteomic analysis

Proteins from serum was extracted and prepared as previously described [75–77], and the extracted peptides was analysed using a RSLC 3000 nanoscale capillary LC followed by qTOF mass spectrometry (5600 Triple-TOF, Sciex). Sequential window acquisition of all theoretical spectra (SWATH) was used to profile all proteins in each sample using a data-independent acquisition method [78]. ProteinPilot™ was used for protein identification and quantitation, as well as visualising peptide-protein associations and relationships.

### Choline extraction

Serum samples were analysed using tandem mass spectrometry (LC-MS/MS) and a multiple-reaction monitoring (MRM) methodology. Five microliters of serum was extracted with 90 μL of an organic solution (10% methanol and 90% acetonitrile), containing the deuterium-labelled internal standard (IS, D9-Cho at 10 μg/mL). This resulted in precipitation of proteins, which were removed by filtration with a Millex 0.45-μm filter followed by centrifugation for 2 min at 6000*g*. The mass transitions used to measure the analytes are choline (mass transition *m/z* 104 → 60) and D9-choline (mass transition *m/z* 113 → 69). A QTRAP 5500 triple-quadrupole mass spectrometer (AB Sciex, Warrington, Cheshire, UK) with ESI ion source was used for data acquisition. Separation of analytes was performed in an Acquity UPLC-MS/MS (Waters, Hertfordshire, UK), with a binary pump system at a flow rate of 0.3 mL/min, connected to the mass spectrometer. The injection volume was 10 μL. Samples were separated using a Cogent 100 mm × 2.1 mm, 4 μm Diamond Hydride silica column (Microsolv Technologies, NJ, USA) and a linear gradient from 65% buffer B (0.1% formic acid in Acetronitrile) and 35% buffer A (0.1% formic acid in water) to 35% buffer B over 7 min. Analyst software (AB SCIEX) was used for HPLC system control, data acquisition and data processing.

### Statistics

The data were analysed using various statistical models. All data were analysed for normal distribution within each experimental group using the Shapiro–Wilk normality test. Linear regression and correlation analysis based on Excel (Microsoft Office 10) built-in functions with interval of confidence and testing of the correlation coefficients were performed according to standard procedures. The SAS software was used to fit the generalised linear model (Microsoft Office 10). Normally distributed data were analysed by ANOVA or *t* tests, as appropriate. Where data were not normally distributed, non-parametric tests were used. When appropriate, *P* values were adjusted for multiple comparisons. Data are presented as histograms (means ± standard error) or box and whisker plots (boxes indicate the 25th and 75th percentiles; whiskers display the range; and horizontal lines in each box represent the median). Regression and correlation coefficients are given with the intervals of confidence (*p* = 0.05). Statistical analyses were performed using Sigma Plot software (v 11.0) (Systat Software Inc., London, UK) and Prism software (GraphPad, USA). *P* value < 0.05 was considered statistically significant.

## Supplementary information

**Additional file 1: Fig. S1.** Ambulatory activity of WT and *Phospho1*^*−/−*^ mice. **(a)** Total activity, **(b)** fast activity, **(c)** slow activity, **(d)** total static counts, **(e)** fast static counts, **(f)** slow static counts, **(g)** total mobile counts, **(h)** fast mobile counts, **(i)** slow mobile counts, **(j)** active time, **(k)** static time **(l)** mobile time, **(m)** front to back, **(n)** inactive time, **(o)** distance travelled. Data are represented as mean ± S.E.M (*n* = 6 replicates). *p < 0.05.

**Additional file 2: Fig. S2.** Fasted glucose levels of 120 day old WT and *Phospho1*^*−/−*^ mice on the control and HFD. Different letters above the error bar show significant difference at p < 0.05.

**Additional file 3: Fig. S3.** μMRI adipose quantification from WT and *Phospho1*^*−/−*^ mice on both a control and HFD. **(a)** Representative reconstructed μMRI scan. Green = subcutaneous adipose tissue, Red = mesenteric adipose tissue, Blue = brown adipose tissue. (**b)** Inguinal WAT (iWAT), mesenteric WAT (mWAT) and brown adipose tissue (BAT) mass determined by μMRI. Results were normalised to body weight (mg/g). Data are represented as mean ± S.E.M (*n* = 3 replicates). Different letters above the error bar show significant difference at p < 0.05.

**Additional file 4: Fig. S4.** Marrow adipose tissue μCT osmium quantification. **(a)** Region-specific quantification of tibial marrow adipose tissue (MAT) volume. Regions include the proximal epiphysis (Prox Epi), the growth plate to the tibia/fibula (Tib/Fib) junction (GP to T/F J) and the tibia/fibula junction to the end of the bone (T/F J to end). **(b)** Representative images of osmium-stained tibiae scanned by μCT. Marrow fat is dark grey and bone is light grey. Data are represented as mean ± S.E.M (n = 3 replicates). * p < 0.05, ** *p* < 0.01.

**Additional file 5: Fig. S5.** Seahorse analysis of WT and *Phospho1*^*−/−*^ osteoblasts. **(a - b)** Oxygen consumption rates (OCR) and extracellular acidification rates (ECAR) using the Seahorse X-24 analyser in WT and *Phospho1*^*−/−*^ primary calvarial osteoblasts cultured in growth media and growth media supplemented with osteogenic differentiation media for 3 days following a Mito Stress Test. Data represented as means ±S.E.M from average of two independent seahorse runs, with *n* = 5 wells per group. **p < 0.01, ***p < 0.001.

**Additional file 6: Table S6.** Osteoblast microarray candidates involved associated with glucose homeostasis. 21 genes from the WT and *Phospho1*^*−/−*^ osteoblast microarray were identified by Ingenuity Pathway Analysis to be associated with glucose homeostasis *p* = 1.04 × 10^− 6^.

**Additional file 7: Table S7.** In Silico analysis of Ingenuity pathways predictions In Silico analysis of genes predicated to be associated with bone and diabetes mellitus.

**Additional file 8: Fig. S8** GeneMANIA network summary predictions GeneMANIA network generated using Ingenuity Pathways Analysis gene predictions. The network highlights potential interactions between *Phospho1* and related osteoblast genes involved in the glucose metabolic process, encompassing; glucose transport, insulin receptor signalling, response to insulin and cellular response to insulin stimulus. Query genes (black) with the exception of Spic and Runx2 which were inputted manually, other genes (grey) were generated by the programme using a large set of inbuilt functional association data. Node size are based on GO terms. Network line colour corresponds to interaction: purple = co-expression, pink = physical interactions, blue = co-localisation, green = shared protein domains orange = predicted, grey = other.

**Additional file 9: Table S9.** In Silico analysis top diseases associated with unique WT HFD and *Phospho1*^*−/−*^ HFD proteins. (CSV 3 kb)

**Additional file 10: Fig. S10.** Gene and protein expression of *Phospho1* mRNA and PHOSPHO1 protein in murine tissue **(a)** RT-qPCR of *Phospho1* in murine tissues, high expression was seen in the gonad and brown adipose tissue (BAT) (**b)** Protein expression of PHOSPHO1 was detectable by western blot in the calvaria and bone. Non-specific binding of the PHOSPHO1 antibody was observed in the pancreas, seen in both WT and *Phospho1*^*−/−*^ pancreatic tissue.

**Additional file 11.** Raw data.

## Data Availability

Data generated or analysed during this study are included in this published article and its supplementary information files or available from the corresponding author on request. All analysed datasets were sourced from the authors. Raw data can be found in Additional file 11.

## Abbreviations

PHOSPHO1: Phosphatase Orphan 1
ANOVA: Analysis of variance
BAT: Brown adipose tissue
CD: Control diet
*Esp*: Embryonic stem cell phosphatase
EV: Empty vector
FCCP: Protonophoric uncoupler
GLA13-OCN: Carboxylated osteocalcin
GLU13-OCN: Undercarboxylated osteocalcin
GLU-OCN: Uncarboxylated osteocalcin
GSIS: Glucose stimulate insulin secretion
GTT: Glucose tolerance test
gWAT: Gonadal white adipose tissue
HbA1c: Glycated haemoglobin
HFD: High fat diet
HOMA-IR: Homeostatic Model Assessment of Insulin Resistance
IPA: Ingenuity Pathway Analysis
ITT: Insulin tolerance test
iWAT: Inguinal white adipose tissue
LC-MS/MS: Liquid chromatography with tandem mass spectrometry
mWAT: Mesenteric white adipose tissue
nSMase2: Neutral sphingomyelinase 2
OCN: Osteocalcin
OE: Overexpressing vector
OST-PTP: Osteotesticular protein tyrosine phosphatase
pCho: Phosphocholine
Pi: Inorganic phosphate
RER: Respiratory exchange rate
SWATH: Sequential window acquisition of all theoretical spectra
*Ucp1*: Uncoupling protein 1
WAT: White adipose tissue
WT: Wild-type
