# Supplementary figures and images for: PHOSPHO1 is a skeletal regulator of insulin resistance and obesity
Source: BMC Biol. 2020 Oct 22;18:149. doi: 10.1186/s12915-020-00880-7 (PMC7584094; doi:10.1186/s12915-020-00880-7)

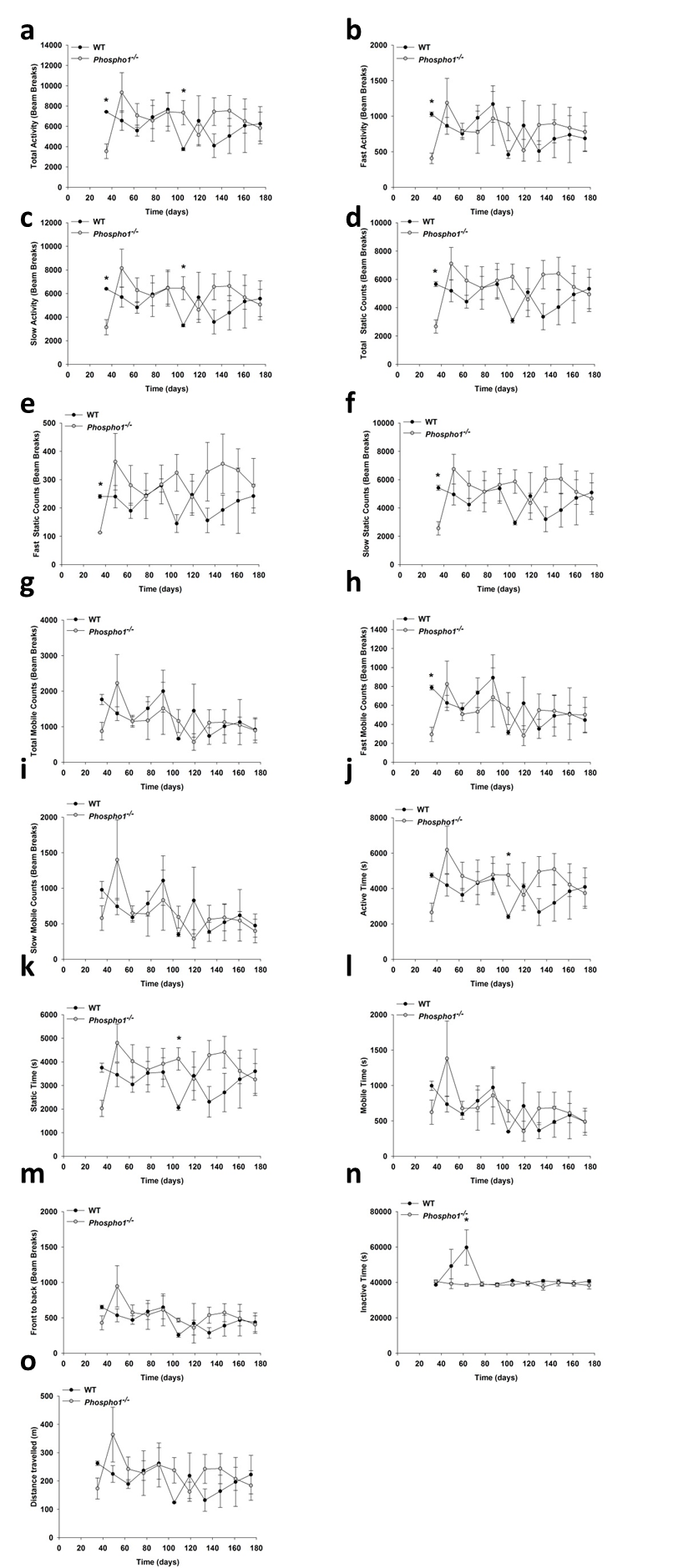

Supplement: Supplementary file 1 — Additional file 1: Fig. S1. Ambulatory activity of WT and Phospho1−/− mice. (a) Total activity, (b) fast activity, (c) slow activity, (d) total static counts, (e) fast static counts, (f) slow static counts, (g) total mobile counts, (h) fast mobile counts, (i) slow mobile counts, (j) active time, (k) static time (l) mobile time, (m) front to back, (n) inactive time, (o) distance travelled. Data are represented as mean ± S.E.M (n = 6 replicates). *p < 0.05. [file 12915_2020_880_MOESM1_ESM.png]

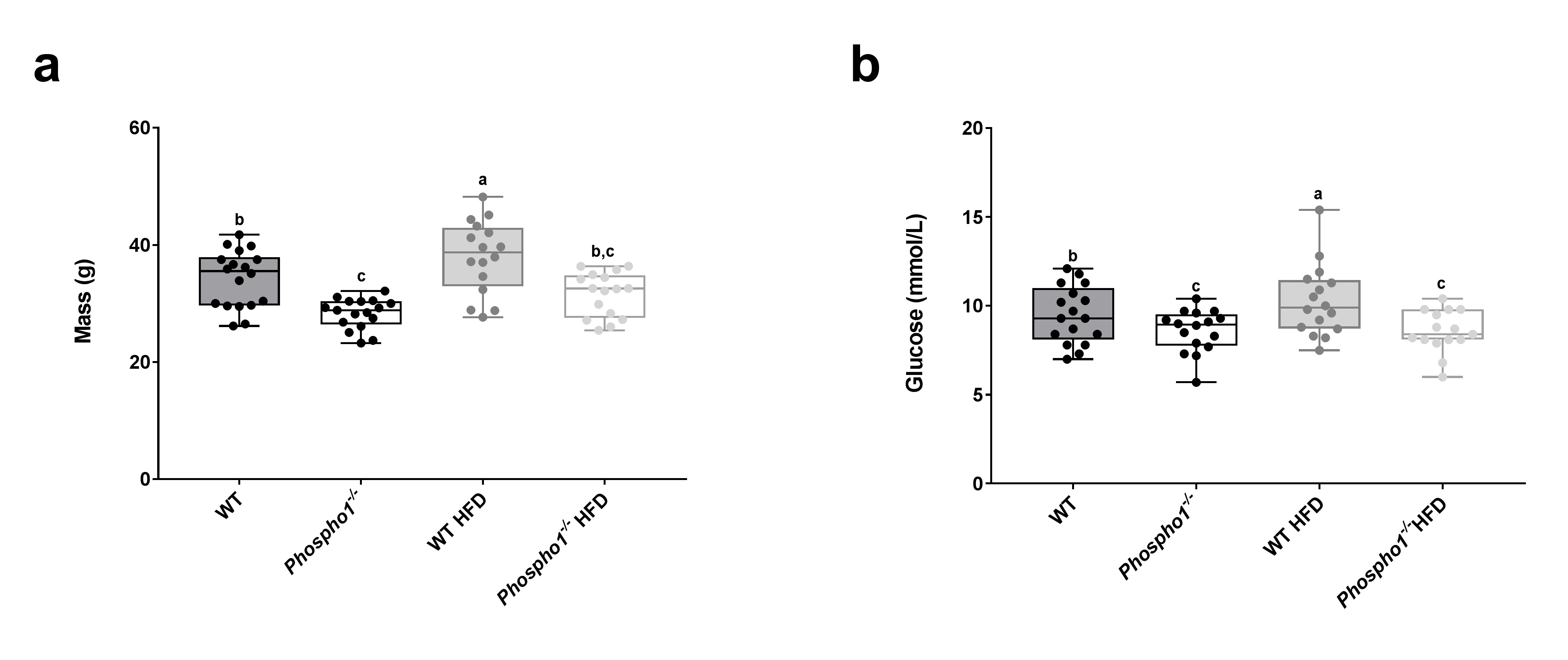

Supplement: Supplementary file 2 — Additional file 2: Fig. S2. Fasted glucose levels of 120 day old WT and Phospho1−/− mice on the control and HFD. Different letters above the error bar show significant difference at p < 0.05. [file 12915_2020_880_MOESM2_ESM.png]

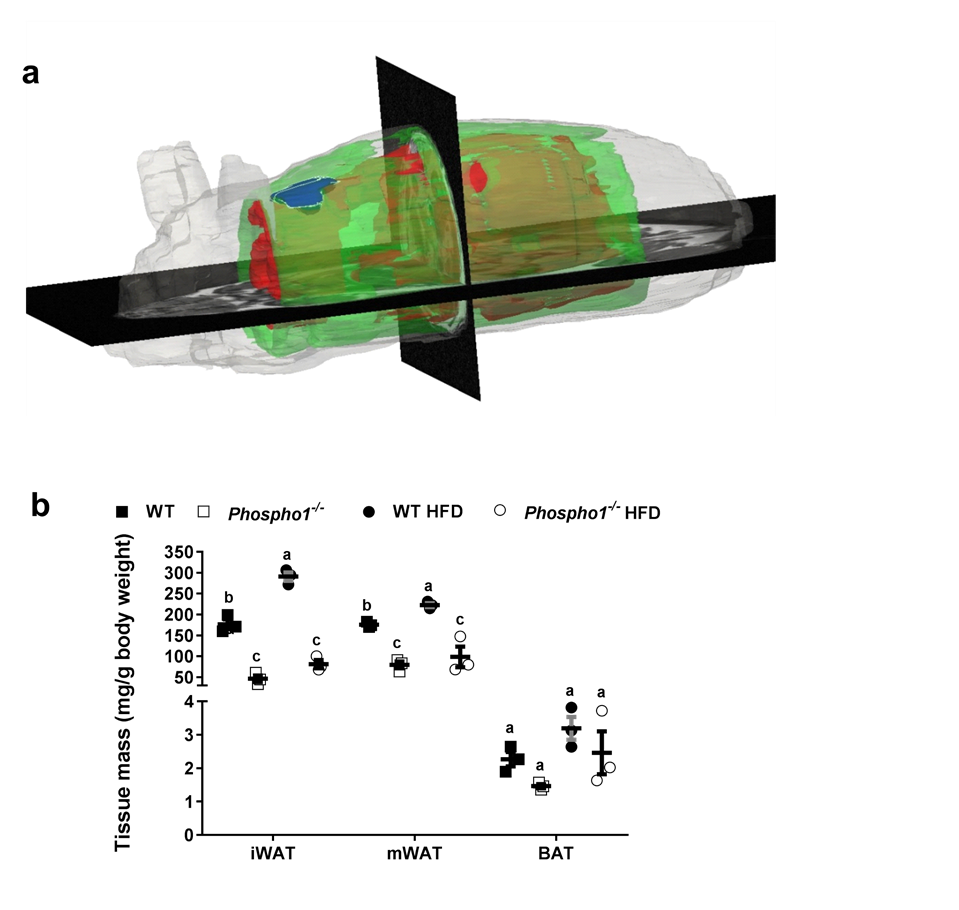

Supplement: Supplementary file 3 — Additional file 3: Fig. S3. μMRI adipose quantification from WT and Phospho1−/− mice on both a control and HFD. (a) Representative reconstructed μMRI scan. Green = subcutaneous adipose tissue, Red = mesenteric adipose tissue, Blue = brown adipose tissue. (b) Inguinal WAT (iWAT), mesenteric WAT (mWAT) and brown adipose tissue (BAT) mass determined by μMRI. Results were normalised to body weight (mg/g). Data are represented as mean ± S.E.M (n = 3 replicates). Different letters above the error bar show significant difference at p < 0.05. [file 12915_2020_880_MOESM3_ESM.png]

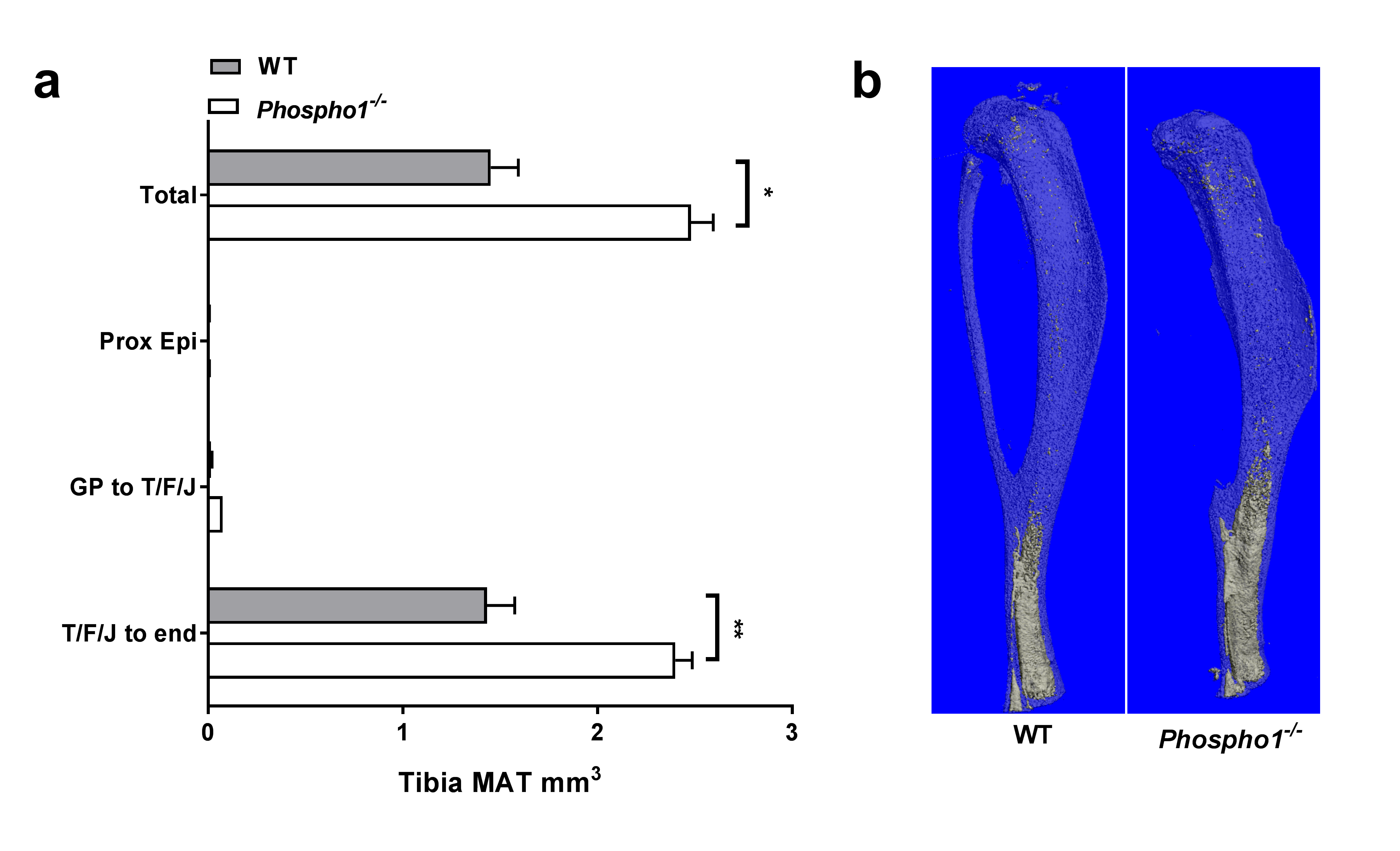

Supplement: Supplementary file 4 — Additional file 4: Fig. S4. Marrow adipose tissue μCT osmium quantification. (a) Region-specific quantification of tibial marrow adipose tissue (MAT) volume. Regions include the proximal epiphysis (Prox Epi), the growth plate to the tibia/fibula (Tib/Fib) junction (GP to T/F J) and the tibia/fibula junction to the end of the bone (T/F J to end). (b) Representative images of osmium-stained tibiae scanned by μCT. Marrow fat is dark grey and bone is light grey. Data are represented as mean ± S.E.M (n = 3 replicates). * p < 0.05, ** p < 0.01. [file 12915_2020_880_MOESM4_ESM.png]

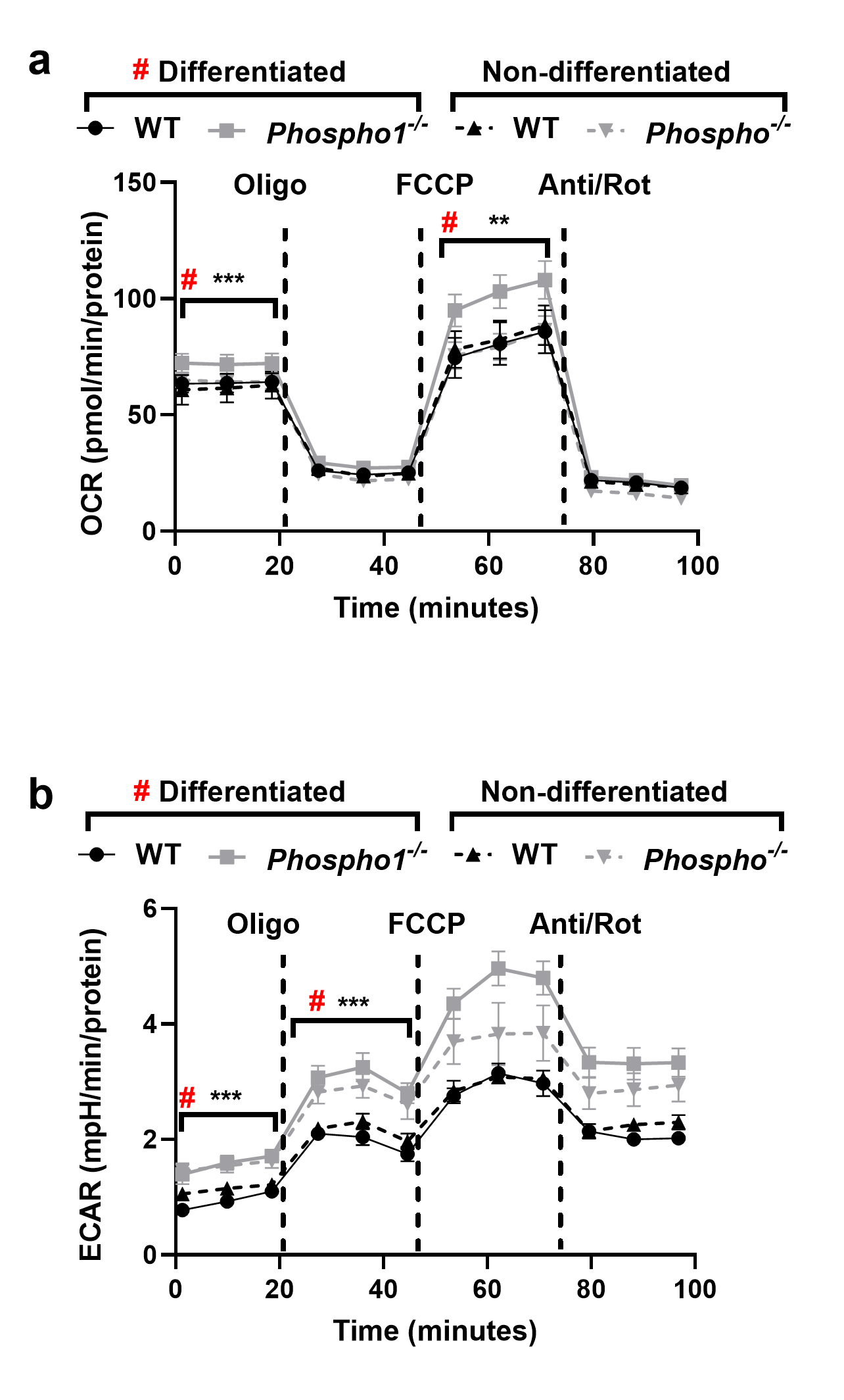

Supplement: Supplementary file 5 — Additional file 5: Fig. S5. Seahorse analysis of WT and Phospho1−/− osteoblasts. (a - b) Oxygen consumption rates (OCR) and extracellular acidification rates (ECAR) using the Seahorse X-24 analyser in WT and Phospho1−/− primary calvarial osteoblasts cultured in growth media and growth media supplemented with osteogenic differentiation media for 3 days following a Mito Stress Test. Data represented as means ±S.E.M from average of two independent seahorse runs, with n = 5 wells per group. **p < 0.01, ***p < 0.001. [file 12915_2020_880_MOESM5_ESM.png]

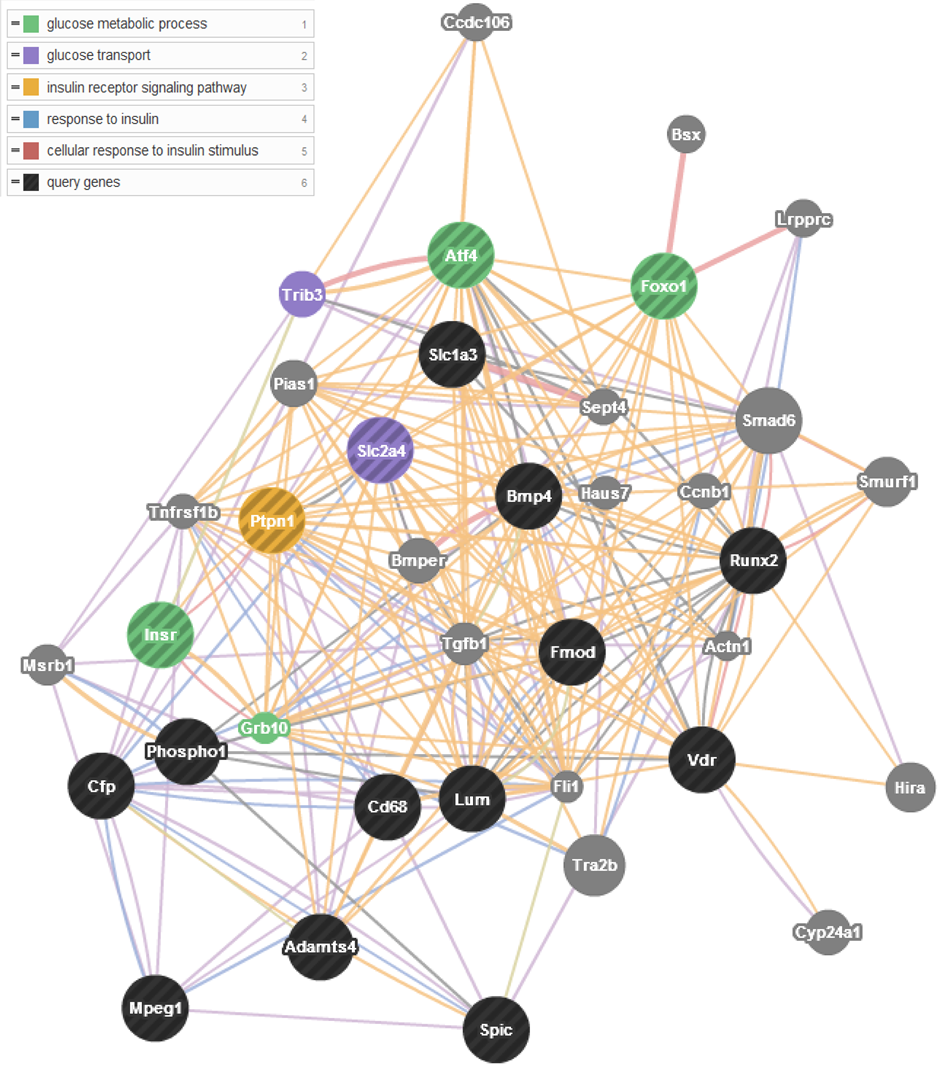

Supplement: Supplementary file 8 — Additional file 8: Fig. S8 GeneMANIA network summary predictions GeneMANIA network generated using Ingenuity Pathways Analysis gene predictions. The network highlights potential interactions between Phospho1 and related osteoblast genes involved in the glucose metabolic process, encompassing; glucose transport, insulin receptor signalling, response to insulin and cellular response to insulin stimulus. Query genes (black) with the exception of Spic and Runx2 which were inputted manually, other genes (grey) were generated by the programme using a large set of inbuilt functional association data. Node size are based on GO terms. Network line colour corresponds to interaction: purple = co-expression, pink = physical interactions, blue = co-localisation, green = shared protein domains orange = predicted, grey = other. [file 12915_2020_880_MOESM8_ESM.png]

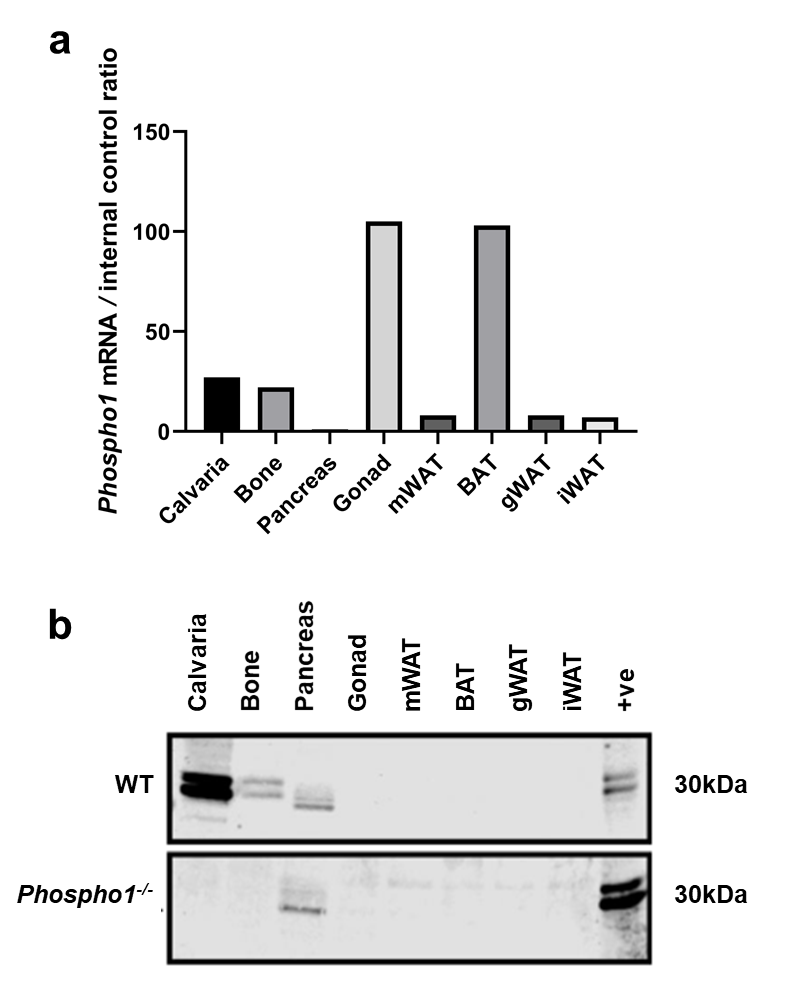

Supplement: Supplementary file 10 — Additional file 10: Fig. S10. Gene and protein expression of Phospho1 mRNA and PHOSPHO1 protein in murine tissue (a) RT-qPCR of Phospho1 in murine tissues, high expression was seen in the gonad and brown adipose tissue (BAT) (b) Protein expression of PHOSPHO1 was detectable by western blot in the calvaria and bone. Non-specific binding of the PHOSPHO1 antibody was observed in the pancreas, seen in both WT and Phospho1−/− pancreatic tissue. [file 12915_2020_880_MOESM10_ESM.png]
